# Big Five traits and cyberbullying severity as predictors of cyberbystander behavior among adults in the U.S.

**DOI:** 10.1186/s40359-026-05073-5

**Published:** 2026-07-17

**Authors:** Haojian Li, Brittany Wheeler, Deborah Hall, Yasin Silva

**Affiliations:** 1 Department of Communication, University of California, Davis, CA USA; 2 Department of Communication, University of California, Santa Barbara, CA USA; 3 School of Social and Behavioral Sciences, Arizona State University, Glendale, AZ USA; 4 Department of Computer Science, Loyola University Chicago, Chicago, IL USA

**Keywords:** Cyberbullying, Cyberbystander, Personality, Social Media

## Abstract

**Background:**

Whereas correlates of cyberbullying have been studied extensively, there has been comparatively less work examining predictors of the different ways in which bystanders to cyberbullying might respond. Adopting a person-situation interaction approach, this study investigated the extent to which the Big Five personality traits and severity of cyberbullying interactively predict the likelihood of different cyberbystander behaviors.

**Methods:**

Adults in the U.S. (*N* = 303) took part in an online survey in which they were presented with a series of nine simulated social media interactions in the form of screenshots that involved exchanges between two social media users. Each screenshot depicted one of three distinct levels of cyberbullying severity: none, low severity, and high severity. For each screenshot, participants were asked to report the likelihood that they would respond in a range of ways as a bystander, including remaining a passive observer, confronting a bully, reinforcing a bully, supporting a victim, and flagging or reporting a post. Participants then completed a measure of the Big Five personality traits.

**Results:**

Regardless of cyberbullying severity, participants were significantly more likely to indicate that they would remain a passive observer in response to the depicted social media interactions than any other cyberbystander behavior. Informative two-way interactions did, however, emerge between cyberbullying severity and cyberbystander behavior and between these variables and Big Five traits across a series of mixed effects models. A significant three-way interaction emerged for agreeableness, such that participants higher in agreeableness reported a greater likelihood of bystander action in response to high severity cyberbullying than those with moderate or lower levels of agreeableness.

**Conclusions:**

This research offers support for the predictive value of both individual differences in the Big Five personality traits and cyberbullying severity for understanding diverse forms of cyberbystander behavior.

**Supplementary Information:**

The online version contains supplementary material available at https://doi.org/10.1186/s40359-026-05073-5.

Mirroring the rise in online communication, smartphones, and social media use have been increases in the prevalence of cyberbullying [1, 2], which is broadly defined as "using information and communication technologies (ICT) to repeatedly and intentionally harm, harass, hurt, and/or embarrass a target" [3]. In recent data from the Cyberbullying Research Center, more than half (i.e., 58%) of teens indicated that they had been the victim of cyberbullying at some point in their life, with nearly one in three (32.7%) teens reporting that they had experienced cyberbullying within the past 30 days [1]. Crucially, cyberbullying, cyberharassment, and related forms of online incivility are also evident among adult populations [4–6]. The importance of empirical work on cyberbullying is underscored by a well-established link between both cyberbullying victimization and perpetration and adverse mental health. For example, multiple research syntheses and meta-analyses have found that cyberbullying victimization is associated with significantly higher levels of depression, anxiety, self-harm, and suicidal thoughts and behaviors [7–9].

An emerging area of research has focused on cyberbystanders—the individuals who witness cyberbullying interactions [10]. Cyberbystanders can play a pivotal role in the escalation or attenuation of a cyberbullying interaction and its impact on a cyberbullying victim [11, 12]. They can, for example, help reduce the frequency and impact of cyberbullying episodes they witness (e.g., by providing social and emotional support to a victim) or they can exacerbate cyberbullying by choosing not to intervene or by supporting or even joining a bully [13]. In fact, the experience of witnessing cyberbullying has, itself, been associated with mental health repercussions. Prior work has shown that bystanders who witness cyberbullying experience empathic distress, particularly when the cyberbullying is severe [14]. In a study of middle school students, Doumas and Midgett [15] found that witnessing someone else being cyberbullied was associated with significantly greater depression, anxiety, and somatic symptoms (e.g., aches, pains). In a notable longitudinal investigation, Tian and colleagues [16] examined bystander behavior trajectories for traditional and cyberbullying across three time points. Their findings revealed complex associations between pro-cyberbullying behaviors and consistent defending behaviors, on one hand, and indicators of psychosocial adjustment, including subjective well-being, internalizing problems, and conduct problems, at 6- and 12-month follow-up. Together, these and other studies point to the importance of studying bystander behavior in the context of social media interactions [17].

Several vital open questions remain, including the extent to which the different ways cyberbystanders may respond to cyberbullying can be meaningfully predicted by characteristics of the bystanders or the cyberbullying situation. The core aim of the present study, which was part of a broader exploratory investigation of cyberbystander behavior carried out as part of the lead author’s master’s thesis [18], was to examine whether the likelihood of performing a range of cyberbystander behaviors might vary as a function of Big Five personality traits and the severity of a cyberbullying interaction. In doing so, we adopt a person-situation interaction approach, which recognizes the interplay between more stable and enduring individual differences (e.g., personality traits) and situational determinants of behavior [19]. Specifically, it emphasizes that the effects of personality depend on the situation and that, conversely, situational factors can elicit different behavioral responses depending on people’s internal traits [20, 21]. We believe the interaction of more stable personality traits with a situational factor like the severity of cyberbullying is important to study from both a theoretical and a practical standpoint.

## How do cyberbystanders respond?

Prior research has found that cyberbystander behavior can be categorized into qualitatively distinct categories. To illustrate, research by Olenik-Shemesh and colleagues [22] investigated socio-emotional predictors of teens’ reports of whether they remained a passive observer or actively intervened—either directly (e.g., by helping a victim) or indirectly (e.g., by telling an adult)—after witnessing someone else being cyberbullied. Others have distinguished between positive and negative cyberbystander behaviors. Jeyagobi and colleagues [13] performed a systematic review of studies investigating negative cyberbystander behaviors, in which they identified distinct types of negative bystander behaviors, including passively standing by, supporting or joining a bully, and aggressing against a bully. Relatedly, some prior research has sought to discern cyberbystander behaviors that are aggressive from those that are more constructive [23, 24]. Moxey and Bussey [23], for instance, developed the Styles of Bystander Intervention Scale to measure the extent to which teens who witness cyberbullying take retaliatory action against a bully (i.e., an aggressive response) or enact a more constructive approach to intervention (e.g., telling a bully to stop, comforting a victim). Sarimento and colleagues [24] identified three types of bystanders—(1) passive outsiders, who remain inactive when witnessing cyberbullying; (2) defenders of cybervictims, who stand up and offer support to the victim; and (3) reinforcers of cyberbullying, who side with the cyberbullying perpetrator and indirectly harm the victim—and developed a scale for assessing these cyberbystander behaviors in both face-to-face and online contexts (i.e., confronting a cyberbully in-person versus online).

In some instances, bystanders may circumvent direct confrontation with bullies, opting instead to extend emotional support to victims either in the comments section of a social media post or through direct messages in instances of cyberbullying. Along these lines, Moxey and Bussey [23] pointed out a potentially meaningful distinction between constructive approaches that involve interacting with a bully (i.e., bully-focused action) and constructive approaches that involve interacting with a victim (i.e., victim-focused action). When validating their Styles of Bystander Intervention Scale, they did not find that this more nuanced categorization of constructive interventions loaded onto separate factors, yet it stands to reason that the likelihood that a bystander will intervene may depend on factors shaping their willingness to interact with a victim versus a bully.

Finally, as touched on in the work of Olenik-Shemesh and colleagues [22], cyberbystander action can differ with respect to how direct or indirect the behavior is. There is, in fact, research indicating that indirect forms of cyberbystander intervention are more common [25, 26]. In an experiment by Dillon and Bushman [25], indirect intervention was measured through participants’ ratings of an ostensible cyberbully’s performance on a task and of the task itself, as well as participants’ communication with the experiment administrator about the cyberbullying incident they had witnessed. These behaviors were considered indirect, in that they did not entail interacting directly with the bully or victim. In a study by DiFranzo and colleagues [26], indirect responses to cyberbullying, which involved flagging a post, reporting an ostensible cyberbully to platform administrators, or blocking a user who had cyberbullied others, were found to be the most common type of cyberbystander intervention.

Drawing on this previous research, we chose distinct types of cyberbystander behaviors to investigate in the present study that capture a combination of prosocial actions that might ultimately benefit a victim (e.g., confronting a bully, providing support to a victim, flagging or reporting cyberbullying content), antisocial actions that might exacerbate the bullying (e.g., reinforcing a bully), and bystander inaction (i.e., remaining a passive observer). These behaviors were chosen on the basis of a holistic assessment of the literature, but also because of the nuanced ways each might be differentially associated with more enduring personality traits of witnesses to cyberbullying and situational factors such as cyberbullying severity.

## Big Five traits and cyberbystander behavior

Previous research has examined personality as a predictor of cyberbystander behavior, with much of the prior work focusing on individual differences in empathy, moral disengagement, the Dark Triad traits, and self-control. For instance, cognitive and affective empathy, which enable an individual to experience another's thoughts or emotions, have been recognized as central elements and primary motivators in bystanders' victim-support behaviors [27–29]. Other research has found that moral disengagement—cognitive mechanisms that enable individuals to circumvent internalized moral standards and thus behave immorally without experiencing distress [30]—not only predicts negative cyberbystander behaviors [31] but also mediates the associations between emotional and cognitive empathy, on one hand, and the cyberbystander roles of reinforcer, defender, and outsider, on the other [10]. Perhaps not surprisingly, the Dark Triad traits—psychopathy, narcissism, and Machiavellianism [32]—have been positively associated with the internet trolling behaviors of cyberbystanders [33] and negatively associated with supportive cyberbystander behaviors [14].

In contrast, the Big Five personality traits—the core traits of extroversion, agreeableness, conscientiousness, neuroticism, and openness identified in the widely-accepted five-factor model of personality [34, 35]—have received comparatively less empirical attention as potential predictors of cyberbystander behavior. There have, however, been a number of studies examining the Big Five traits in relation to cyberbullying perpetration and victimization, highlighting the potential value of examining the Big Five traits as predictors of cyberbystander behavior. For instance, given the role of empathy in eliciting prosocial behaviors aimed at assisting or supporting a cyberbullying victim [27–29], it is not surprising that cyberbystanders’ own prior experience as a victim of cyberbullying is associated with an increased likelihood of intervening to assist other cyberbullying victims [36]. Indeed, in a meta-analysis averaging across seven independent studies, Sobol and colleagues [37] found evidence of a robust positive correlation between cyberbullying victimization and the likelihood of adolescent cyberbystanders coming to the defense of other cyberbullying victims—a finding that was also supported in the meta-analysis by a reliable positive link between cyberbullying victimization and empathic skills. In a systematic review by Jeyagobi and colleagues [13], cyberbystanders’ prior experience as a perpetrator of cyberbullying was found to be positively correlated with the likelihood of joining or reinforcing a cyberbully [13]. Thus, we briefly review prior work that sheds light on the association of each Big Five trait with cyberbullying victimization, cyberbullying perpetration, and, if available, cyberbystander behavior, specifically, or prosocial behavior, in general.

### Extroversion

Extroversion, a trait characterized by higher levels of sociability, assertiveness, energy, and a tendency to seek stimulation and positive social interactions [34, 35, 38], has been linked in some prior research to both cyberbullying victimization and perpetration. For instance, in research by Peluchette and colleagues [39], higher extroversion was associated with increased cyberbullying victimization [39], and in a study by Adamopoulou and Kokia [40], higher extroversion corresponded with an increased likelihood of cyberbullying perpetration [40]. Other studies, however, have failed to identify a reliable association between extroversion and cyberbullying (e.g., [41])—a finding also reported in a recent meta-analysis by Sun and colleagues [42]. Specifically, averaging across 20 different studies (and 33 distinct effect sizes) that examined extroversion and cyberbullying victimization and across 15 different studies (and 24 distinct effects) examining extroversion and cyberbullying perpetration, no reliable correlation emerged between extroversion and either aspect of cyberbullying. In one of the few studies we are aware of that examined Big Five personality traits in relation to being a cyberbystander, Zhou and colleagues [41] found that extroversion was not associated with the extent to which participants indicated they had observed others experiencing cyberbullying. In light of this, we have no *a priori* hypotheses regarding how extroversion may differentially predict cyberbystander behaviors that either support or benefit a victim or reinforce a bully. Yet, one interesting possibility, given prior research establishing extroversion as the Big Five trait most strongly and positively associated with general social media use and the size of one’s social media networks [43], is that cyberbystanders who are high in extroversion may be less likely to remain a passive observer upon witnessing a cyberbullying interaction.

### Agreeableness

Agreeableness, which is characterized by a focus on maintaining social harmony, cooperation, and a prosocial orientation toward others [34, 35, 38], has been associated in prior research with an increased likelihood of cyberbullying victimization and a decreased likelihood of cyberbullying perpetration. Escortell and colleagues [44], for example, found that agreeableness was a positive predictor of cyberbullying victimization, and Van Geel and colleagues [45] found that individuals higher in agreeableness were less likely to be involved in cyberbullying perpetration. Notably, in the meta-analysis by Sun and colleagues [42], a robust negative correlation emerged between agreeableness and both victimization and perpetration, averaging across 30 and 28 effects, respectively. In the study by Zhou et al. [41], agreeableness was associated with a decreased likelihood of having observed others being cyberbullied. Given the nature of agreeableness and a well-established link between agreeableness and empathy [46], there are compelling reasons to anticipate that agreeableness may be a meaningful predictor of specific types of cyberbystander behavior. To illustrate, in research by Habashi et al. [47], agreeableness was the only Big Five trait that robustly predicted an increased likelihood of prosocial behavior across three studies, with this association mediated by increased empathy. Moreover, agreeableness was found in a meta-analysis by Kline et al. [48] to be one of two Big Five traits (along with openness) that were correlated with increased prosocial behavior (e.g., volunteerism, making donations). It thus stands to reason that agreeableness may correspond with an increased likelihood of engaging in behaviors aimed at supporting a cyberbullying victim. One more nuanced possibility is that cyberbystanders who are high in agreeableness may be motivated to actively intervene when they witness others being cyberbullied (rather than remaining a passive observer), but may choose to do so by interacting with the victim or reporting a post rather than confronting a bully.

### Openness

Research on openness, a trait reflecting higher levels of curiosity, imagination, and a preference for novelty and creativity [34, 35, 38], has revealed mixed findings in relation to cyberbullying. While some scholars have found individuals' levels of openness to be a protective factor [44], other scholars have found that it is also predictive of increased levels of cyberbullying victimization. Whereas Zhou and colleagues found that openness was significantly negatively correlated with cyberbullying victimization, perpetration, and the likelihood of having witnessed others being cyberbullied [41], openness was not reliably correlated with cyberbullying victimization in the meta-analysis by Sun et al. [42]. That is, averaging across 18 studies (and 29 distinct effects), the bivariate correlation between openness and cybervictimization was near zero. In contrast, a significant (albeit small) negative correlation between openness and cyberbullying perpetration emerged across 13 studies (and 22 effects). In the broader context of prosocial behavior, openness has been associated with an increased tendency toward prosocial behavior [48], suggesting that it may predict a greater likelihood of engaging in victim-supporting cyberbystander behavior. We, therefore, anticipate that openness will be associated with an increased likelihood of supporting a cyberbullying victim, but have no *a priori* predictions regarding the extent to which it will predict other types of cyberbystander behavior.

### Neuroticism

Neuroticism, defined as a tendency toward elevated stress reactivity evidenced in heightened negative emotions [34, 35, 38], has been associated with an increased likelihood of both cyberbullying victimization [42] and perpetration [48, 49], with slightly more robust support for a link with cyberbullying perpetration [42]. Notably, however, Zhou and colleagues found that neuroticism predicted a greater likelihood of having witnessed others being cyberbullied only and was uncorrelated with one’s own likelihood of being a victim or perpetrator of cyberbullying [41]. It is thus unclear to what extent neuroticism may predict different cyberbystander behaviors. One interesting and more nuanced pattern of potentially relevant findings comes from the research by Habashi et al. [47], in which neuroticism predicted an increased tendency toward prosocial behavior. Across multiple studies, neuroticism was actually more strongly associated with experiences of personal distress as a bystander (i.e., a negatively-valenced empathic response) than with the likelihood of taking prosocial action to reduce the distress. It is thus possible that for bystanders who are higher in neuroticism, witnessing others being cyberbullied may elicit relatively stronger negative emotional responses, including empathic concern and personal distress, but this elevated stress response may not translate into an increased likelihood of active bystander intervention.

### Conscientiousness

Conscientiousness, characterized by a preference for organization, self-discipline, reliability, and a tendency to be goal-directed [34, 35, 38], has been linked with a decreased likelihood of both cyberbullying victimization and perpetration. In the meta-analysis by Sun and colleagues [42], these findings emerged across 19 studies (30 effects) and 15 studies (24 effects), respectively, with a slightly stronger negative correlation between conscientiousness and cyberbullying perpetration. In the study by Zhou and colleagues [41], conscientiousness was uncorrelated with cyberbullying victimization, perpetration, and likelihood of having witnessed others being cyberbullied. In the research of Habashi and colleagues, conscientiousness was associated with higher empathic concern as a bystander but not a robust increase in prosocial behavior. Thus, we have no *a priori* predictions regarding the extent to which conscientiousness may relate to different cyberbystander behaviors.

On the whole, the extant literature suggests that individual differences in the Big Five personality traits predict, to varying degrees, differences in cyberbullying experiences and, therefore, may provide a useful framework for predicting a range of distinct ways in which bystanders to cyberbullying choose to respond. Although less directly relevant to cyberbystander behavior, one novel study that we believe warrants mention comes from Balakrishnan and colleagues [50]. Specifically, their research team extracted features reflecting content that mapped onto each of the Big Five traits from a dataset containing 9,484 tweets crawled from Twitter, a subset of which (i.e., 528 tweets) had been labeled to indicate the role of cyberbullying perpetrator. They found that including information pertaining to extroversion, agreeableness, and neuroticism, in particular, improved an automated model’s ability to correctly identify tweets involving a cyberbully. These findings converge with much of the prior research reviewed above, suggesting that some Big Five traits (e.g., extroversion, agreeableness, neuroticism) are more robust predictors of cyberbullying experiences than others (e.g., conscientiousness) and theorizing regarding how specific traits might uniquely relate to distinct cyberbystander behaviors.

We also note that prior work by Janošová and colleagues [51] found that agreeableness and neuroticism were positively related to defending behavior in the context of traditional bullying in school, and Pronk and colleagues [52] found that agreeableness was a significant predictor of a greater likelihood of remaining a passive observer and defending a victim among fifth and sixth-grade students who witnessed traditional bullying. Nevertheless, there are several crucial ways in which traditional bullying in school settings differs from cyberbullying on social media [7, 53], underscoring the need for empirical work that more directly examines the Big Five traits as predictors of cyberbystander behavior.

## Cyberbullying severity

Complementing the prior research investigating cyberbystander behavior through the lens of individual differences in internal traits, a number of studies have examined situational factors that contribute to when and how cyberbystanders choose to intervene. Following the bystander intervention model [54]—which delineates a five-step decision-making process that witnesses to emergencies, broadly, engage in when determining whether to remain a passive bystander or intervene to assist a potential victim—previous studies have, for instance, sought to identify situational determinants of the likelihood that cyberbystanders will interpret an online interaction as cyberbullying and experience a sense of personal responsibility to intervene. These have included investigations of the social relationship of a bystander to the individuals involved in the cyberbullying [55, 56], perceived size of the audience viewing the cyberbullying content [26], number of cyberbullies [57], behaviors of other bystanders [11], form of social media content (e.g., original content vs. share/re-post) [57], and characteristics of a social media platform such as platform notification settings [26] and platform features designed to elicit empathy [26, 58].

One situational factor that may be particularly influential in shaping cyberbystander responses is the perceived severity of a cyberbullying interaction. Indeed, prior research has found evidence of a positive correlation between cyberbullying severity and bystanders’ motivation to intervene to provide assistance to the victim [11, 12, 59]. Conversely, bystanders were more likely to engage in behaviors that reinforce aggression if they interpreted the incident as a joke between the parties involved [60]. Indirect evidence of a link between cyberbullying severity and bystander intervention comes from a study by Van Cleemput et al. [31], which found that bystanders were more likely to remain passive observers when they had difficulty interpreting the nature of the incident they were witnessing [31]. In a recent study by Wang [61], higher cyberbullying severity was found to increase cyberbystanders’ empathic distress, anticipated guilt, and the emotional rewards of helping, which motivated both direct and indirect intervention behaviors.

Yet, no prior work, to our knowledge, has employed a person-situation approach to investigate how cyberbystanders’ internal traits may interact with the perceived severity of a cyberbullying instance in predicting different types of cyberbystander behavior. More severe instances of cyberbullying may be more likely to elicit feelings of empathic concern and distress, particularly among cyberbystanders with a greater tendency toward empathic responses, such as individuals higher in agreeableness or neuroticism [46, 47, 59]. At the same time, higher severity cyberbullying may shift the cost–benefit analysis for a cyberbystander— both in terms of potential costs to oneself and potential consequences of failing to provide assistance to a cyberbullying victim, for instance—in different ways depending on specific Big Five traits.

## Present study

The aim of the present study was to investigate the extent to which the Big Five personality traits predict different types of cyberbystander behaviors and, crucially, whether these associations might vary depending on the severity of a cyberbullying interaction. Following previous research [57, 62], our study design involved a series of screenshots depicting ostensible social media interactions. That is, we employed a repeated-measures design in which participants viewed a series of screenshots of ostensible social media sessions depicting interactions involving high-severity cyberbullying, low-severity cyberbullying, and no cyberbullying. After viewing each screenshot, participants indicated how likely they would be to respond in a variety of ways. Participants then completed individual difference scales that included a measure of the Big Five personality traits and provided demographic information.

## Method

### Pilot study

We first conducted a pilot test to confirm the effectiveness of our key experimental materials with a sample of (*N* = 50) participants recruited from Prolific.com. Eligibility criteria included being 18 years old or older, being a U.S. resident, and reporting use of at least one of the following social media platforms: Facebook, Twitter, Snapchat, TikTok, WhatsApp, Instagram, Pinterest, Reddit, or Discord. Specifically, participants completed an online survey during which they viewed a series (presented in randomized order) of 12 screenshots of ostensible Instagram sessions. Four of the screenshots were designed to depict an interaction with no cyberbullying, four depicted an interaction with low severity cyberbullying, and four depicted an interaction with high severity cyberbullying.

**Fig. 1:**
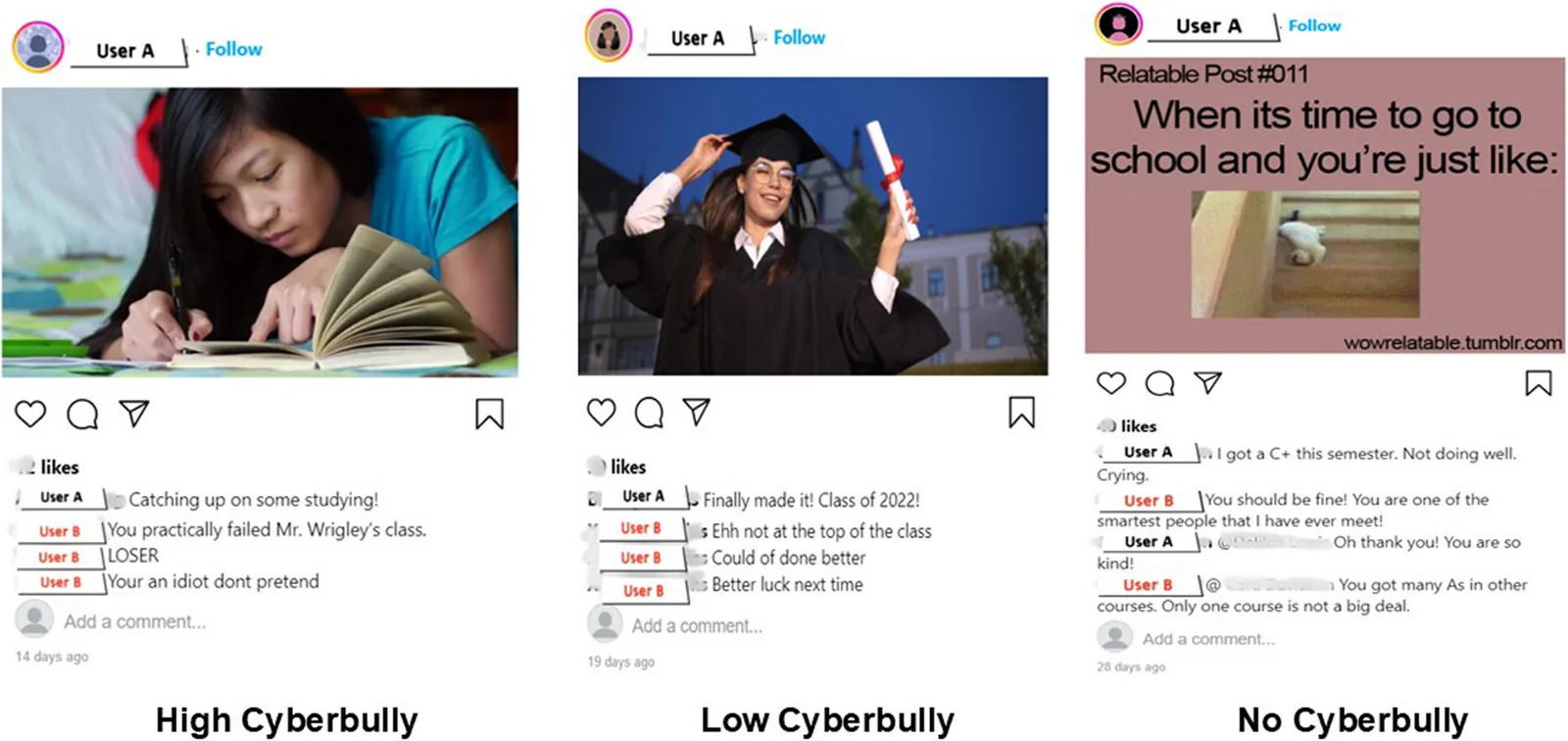
Examples of screenshots for each severity level

As shown in Fig. 1, each screenshot depicted a dialogue between two users, User A (the cyberbullying victim, in interactions involving cyberbullying) and User B (the cyberbullying perpetrator, in interactions involving cyberbullying). The setup involved User A publishing a post and User B leaving comments in response to User A’s initial post. User-specific identifiers appeared to be obscured, with usernames, number of likes and shares, and profile images blurred out. The dialogue followed two specific structures: an alternating pattern of dialogue (User A comment, User B comment, User A comment, User B comment), wherein User A would counterargue against User B's aggressive comments in the cyberbullying conditions, and a non-alternating pattern (User A, User B, User B, User B), in which User A did not respond to User B's comments. Instances of high severity cyberbullying involved verbal attacks on physical appearance or intelligence. Low severity cyberbullying comprised criticisms about attire and gaming skills. Lastly, instances with no cyberbullying involved discussions about topics like travel or food.

After viewing each screenshot, participants were asked: (a) "How positive versus negative would you rate the interaction between the two users communicating in the screenshot above?" (1 = *extremely negative*, 9 = *extremely positive*) and (b) "To what extent do you think the communication between users in this screenshot constitutes or involves cyberbullying?" (1 = *not at all cyberbullying*, 9 = *extremely high degree of cyberbullying*). If participants chose any response other than *not at all cyberbullying* for the second question, they were prompted to answer a third question: (c) "How severe would you consider the cyberbullying in the screenshot to be?" (1 = *not at all severe*, 9 = *extremely severe*).

**Fig. 2:**
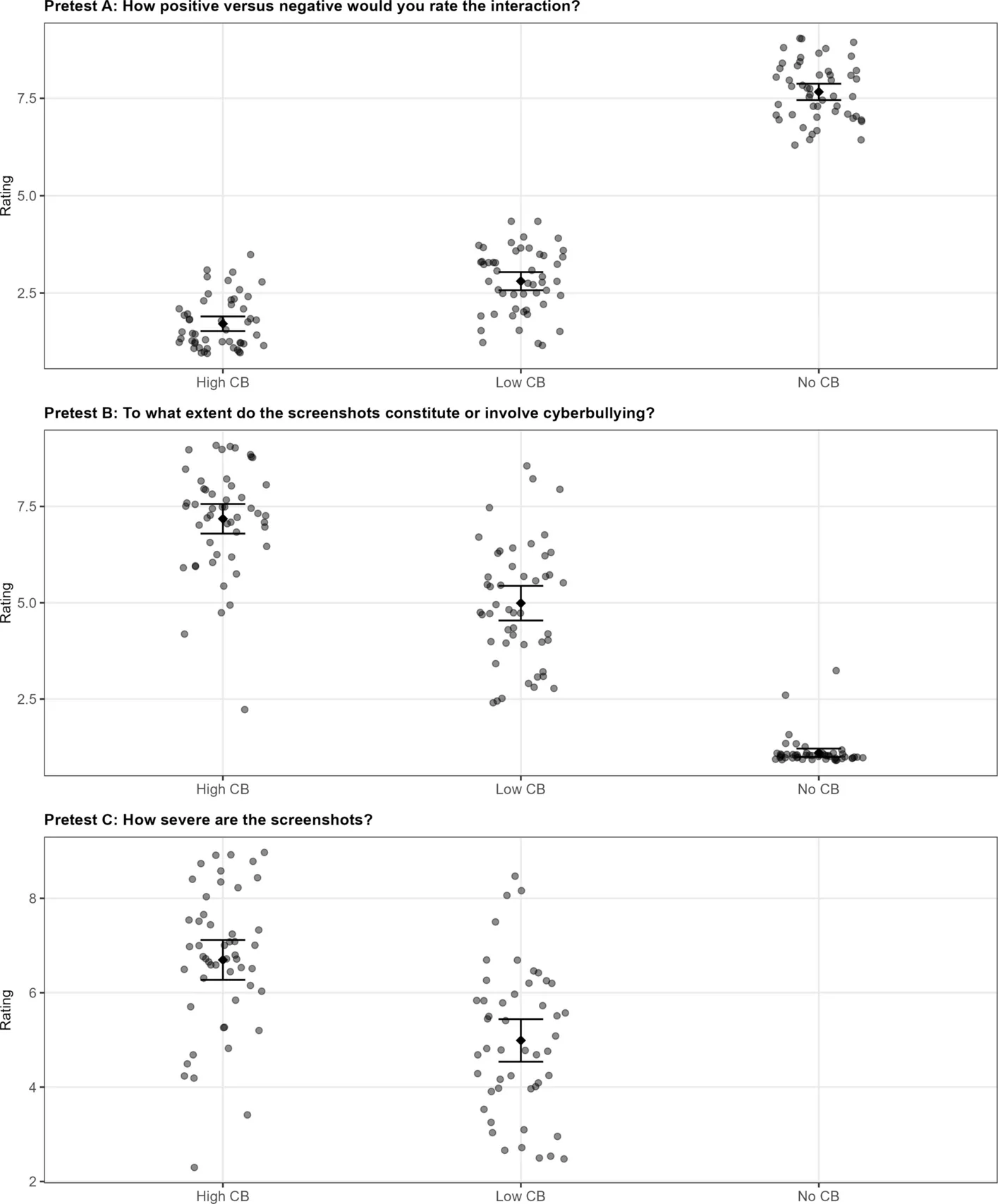
Distribution of individual ratings across cyberbullying severity conditions

An average score for the four screenshots depicting each severity level (no cyberbullying, low severity, high severity) was calculated for the valence of the interaction and the presence of cyberbullying. The distribution of individual ratings across the three severity conditions is presented in Fig. 2.

We then conducted one-way ANOVAs with pairwise comparisons to investigate the effectiveness of the screenshots. The results revealed significant differences in valence, *F*(2, 447) = 2,758.00, *p* < 0.001, *η*_*p*_^2^ = 0.93. and severity of cyberbullying, *F*(2, 447) = 812.40, *p* < 0.001, *η*_*p*_^2^ = 0.78 across the three conditions. In particular, pairwise comparisons indicated that the screenshots intended to depict high severity cyberbullying were rated as being significantly more negative in valence  (*M* = 1.71, *SD* = 0.66) than the screenshots intended to depict low severity cyberbullying (*M* = 2.81, *SD* = 0.82), Welch’s *t*(93) = 7.31, *p* < 0.001, and that the low severity screenshots were rated as being significantly more negative than those depicting no cyberbullying (*M* = 7.67, *SD* = 0.74), Welch’s *t*(97) = 31.09, *p* < 0.001. Additionally, the screenshots intended to depict high severity cyberbullying were rated as significantly higher in degree of cyberbullying (*M* = 7.18, *SD* = 1.35) than the screenshots intended to depict low severity cyberbullying (*M* = 4.79, *SD* = 1.81), Welch’s *t*(90) = 7.48, *p* < 0.001, and the low severity screenshots were rated as significantly higher in degree of cyberbullying than those depicting no cyberbullying (*M* = 1.11, *SD* = 0.38), Welch’s *t*(53) = 14.06, *p* < 0.001. Notably, two of the 12 screenshots (one high severity and one low severity) were determined to be of low quality, due to the deviation of their severity score from the average for their respective severity-level group, and were, therefore, excluded. To maintain uniformity in the number of screenshots across each severity level, we also excluded one screenshot from the non-cyberbullying category, leaving us with three screenshots for each condition to use in the main study.

### Main study

The main study employed a 3 × 5 within-subjects design, in which we manipulated the severity of cyberbullying (none, low, or high) across screenshots and measured participants’ likelihood of engaging in five cyberbystander behaviors. Data were analyzed using linear mixed-effects models to account for repeated observations nested within participants.

### Participants

An a priori power analysis using G*Power [63] indicated that the minimum sample size required to detect a small-medium effect (Cohen’s *f* = 0.175) given the 3 × 5 within-subjects design (with a significance level of *α* = 0.05 and power = 0.90) and employing the conservative approach of treating repeated measurements as independent observations (see Maxwell et al., [64]) was *N* = 237. Because the present investigation was part of a larger empirical study that included additional analyses and to account for potential data loss due to incomplete responses, we ultimately recruited a sample of (*N* = 303) participants from Prolific.com. Participants took part in a ~15-min online survey for which they were compensated $2.00. As with the pilot study, all participants were 18 years or older and U.S. residents, and reported using at least one of the following social media platforms: Facebook, Twitter, Snapchat, TikTok, WhatsApp, Instagram, Pinterest, Reddit, or Discord.

Due to an oversight during the development of the online survey materials, the study did not include any direct checks on participants’ attentiveness. To address this, we tested for potential inattention by regressing each reverse-worded item on the non-reverse items of its scale and flagged participants whose residuals exceeded three standard deviations. This procedure identified three participants with outlying residuals. The inclusion versus exclusion of these three cases from the primary analyses, however, yielded comparable results. We thus retained these three participants and report the results with the full sample.

More than half of the sample identified as female (*n* = 170, 56.1%; male: *n* = 112, 37.0%; other: *n* = 21, 6.9%). Participants ranged in age from 18 to 76 years old (*M* = 39.5, *SD* = 13.9) and predominantly identified as White (*n* = 192, 63.4%; Latino: *n* = 19, 6.3%; Asian: *n* = 29, 9.6%; Black or African American: *n* = 28, 9.2%; Multi-race: *n* = *23*, 7.6%, other: *n* = 11, 3.6%). See Table 1 for additional demographic characteristics of the sample.

**Table 1:**
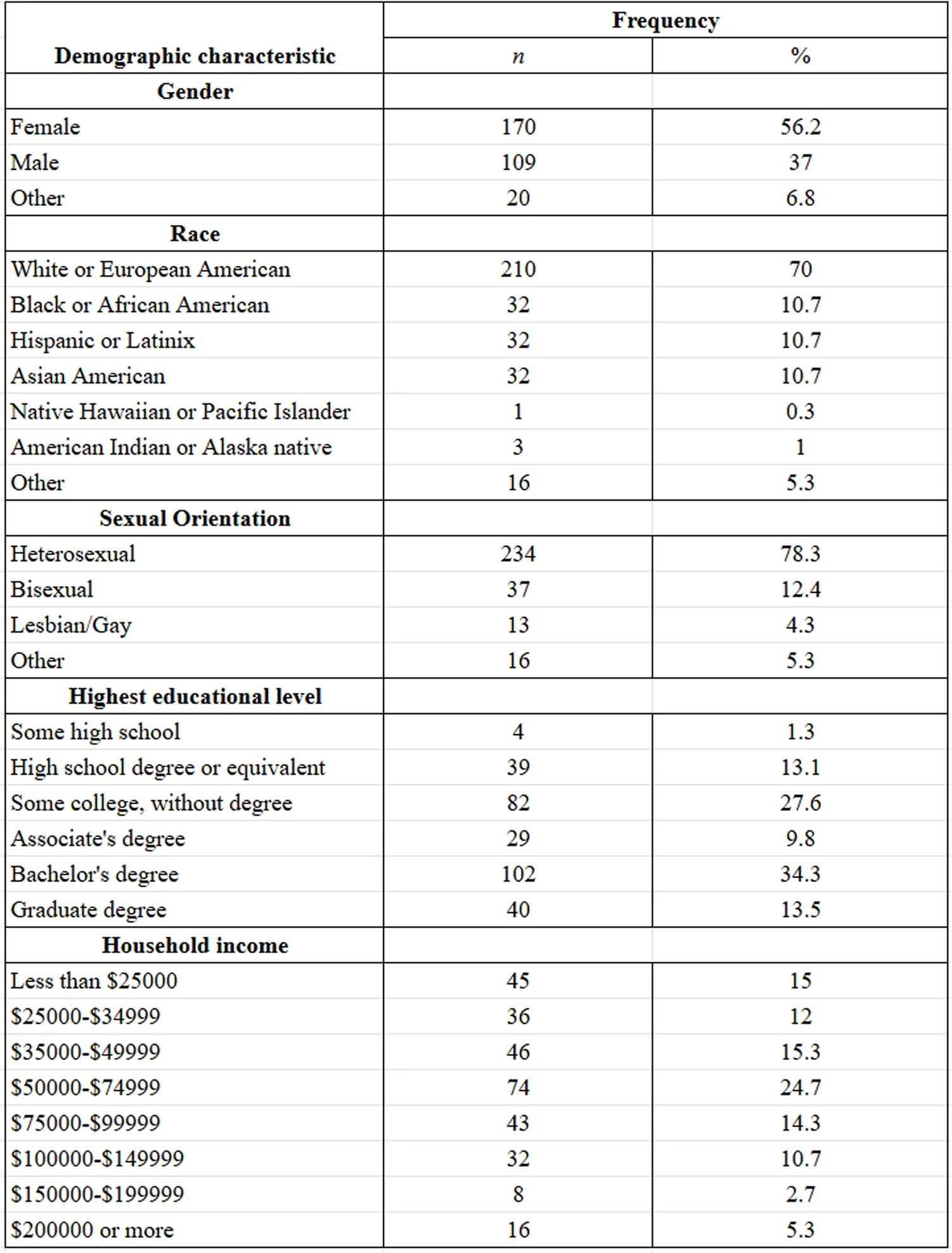
Demographic information for participants

### Materials and measures

#### Cyberbullying screenshots

Each participant viewed a randomized sequence of the nine screenshots depicting ostensible Instagram interactions selected based on pilot testing, with three screenshots presented for each cyberbullying severity level (no cyberbullying, low severity, high severity).

#### Cyberbystander behavior

After viewing each screenshot, participants were asked to indicate the likelihood that they would engage in five specific behaviors in response to the depicted interaction: *remain a* *passive observer*, *confront the bully*, *reinforce the bully*, *support the victim*, and *flag/report the post*. Remaining a passive observer was assessed with one item: “I would scroll past the post without doing anything.” Confronting the bully was assessed with two items: “I would express disagreement with or confront User B, via a direct message”; “I would express disagreement with or confront User B, by posting my own comment or “disliking” one of their comments. Reinforcing the bully was assessed with two items: “I would support or agree with User B, via a direct message”; “I would support or agree with User B, by posting my own comment or by “liking” one of their comments.” Supporting the victim was assessed with two items: “I would support or agree with User A, via a direct message”; “I would support or agree with User A, by posting my own comment or by “liking” one of their comments.” Lastly, reporting/flagging the post was assessed with one item: “I would flag and/or report this post to the social media platform.” Participants rated their likelihood of performing each behavior using a slider ranging from 0 (*0% likelihood*) to 100 (*100% likelihood*).

For each screenshot, mean scores for confronting the bully, reinforcing the bully, and supporting the victim were calculated by averaging participants’ scores on the two relevant items. Across different severity conditions, the three stimuli showed satisfactory consistency in the assessment of each bystander behavior. A composite score for each of the five behaviors was then calculated for each cyberbullying severity level by averaging a participant’s cyberbystander behavior scores across the three screenshots at each severity level, with higher scores reflecting a greater likelihood of performing the respective behavior. Reliability was high for the three multiple-item measures: confronting the bully (mean α = 0.86, range from 0.80 to 0.90 across the three severity levels, *r*_*s*_ = 0.85), reinforcing the bully (mean α = 0.83, range from 0.80 to 0.87 across the three severity levels, *r*_*s*_ = 0.84), and supporting the victim (mean α = 0.83, range from 0.79 to 0.89 across the three severity levels, *r*_*s*_ = 0.86).

#### Big Five personality traits

To measure the Big Five personality traits, we used the BFI Short Form Scale originally developed by Soto & John [65], comprising the factors of extroversion, agreeableness, conscientiousness, neuroticism, and openness. Sample questions included “I am someone who tends to be quiet” (extroversion, reverse-scored); “I am someone who is compassionate, has a soft heart” (agreeableness); “I am someone who is reliable, can always be counted on” (conscientiousness); “I am someone who worries a lot” (neuroticism); “I am someone who is original, comes up with new ideas” (openness). Responses were measured on a 5-point scale from 1 (*strongly disagree*) to 5 (*strongly agree*). A composite variable was computed for each Big Five trait, with each subscale demonstrating sufficient reliability: extroversion (α = 0.79), agreeableness (α = 0.76), conscientiousness (α = 0.85), neuroticism (α = 0.89), and openness (α = 0.82).

#### Sociodemographic information

Participants were asked to indicate their age, ethnicity/race, gender, sexual orientation, highest level of educational attainment, and annual household income. (See Table 1 for demographic response options).

#### Additional measures

The survey included additional individual difference measures not relevant to the present analyses, including scales assessing social dominance orientation, the Dark Triad traits, moral disengagement, and self-control. A full discussion of these measures, analyses, and results can be found in Li [14].

### Analytical approach

In this study, we tested a series of linear mixed-effects models (LMEMs) using the MIXED procedure in IBM SPSS Statistics (Version 29.0.2.0) to account for the variability between individuals while modeling within-person repeated measures across all 15 conditions. Five separate LMEMs were estimated using restricted maximum likelihood, one for each Big Five personality trait (i.e., extroversion, agreeableness, conscientiousness, neuroticism, and openness). Each LMEM was performed with the severity of the cyberbullying depicted (i.e., no cyberbullying, low severity, and high severity), cyberbystander behavior type (i.e., passive observer, confront bully, reinforce bully, support victim, and flag post), and the respective personality trait (standardized) as fixed effects, along with all two-way interactions (i.e., Severity × Behavior Type, Severity × Trait, and Behavior Type × Trait) and the three-way interaction (Severity × Behavior Type × Trait). Participant age, income, and education were included as covariates. Participant ID was specified as a random effect to account for any individual differences in baseline ratings. Given the fully crossed design and the study’s sample size, a diagonal covariate structure was specified for the 15 conditions. To calculate degrees of freedom and generate *p*-values, we utilized Satterthwaite’s method for mixed models [66]. Bonferroni-corrected pairwise comparisons of estimated marginal means were conducted to probe any significant interactions. This is demonstrated in the following formula.

Y_ijk_ ​= β_0​_ + β_1_​S_i_​ + β_2_​B_j_​ + β_3_​E_k_​ + β_4_​(S_i​_×B_j​_) + β_5​_(S_i_​×E_k_​) + β_6_​(B_j_​×E_k_​) + β_7​_(S_i​_×B_j​_×E_k_​) + β_8_​Age_k_​ + β_9_​Income_k​_ + β_10​_ Education_k_​ + u_0k_ ​+ ϵ_ijk_​

For each model, ratings (Y_ijk_​) were modeled as a function of severity (​S_i​_), behavior type (​B_j_), and personality trait (E_k_​), their two-way interactions (e.g., S_i_​ × B_j_) and three-way interaction (S_i_​ × B_j_​ × E_k_), participant-level covariates (e.g., Age_k_​), a random intercept for each participant (u0_k_), and a residual error term (ϵ_ijk_​) [67].

## Results

**Fig. 3:**
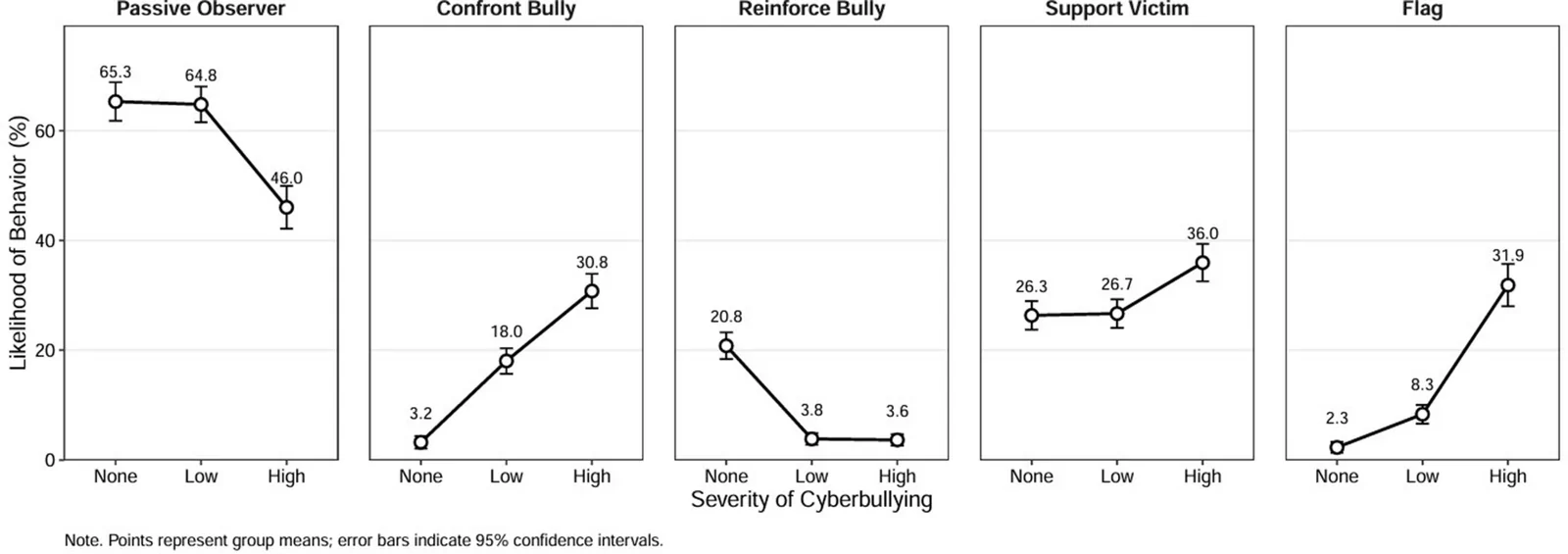
Likelihood of cyberbystander behaviors across severity levels

Table 2 shows the means, standard deviations, and bivariate correlations for the key study variables. A bar graph presenting the likelihood of each cyberbystander behavior across the three severity levels is presented in Fig. 3, which shows the general trends in cyberbystander behavior without taking the Big Five traits into account.

**Table 2:**
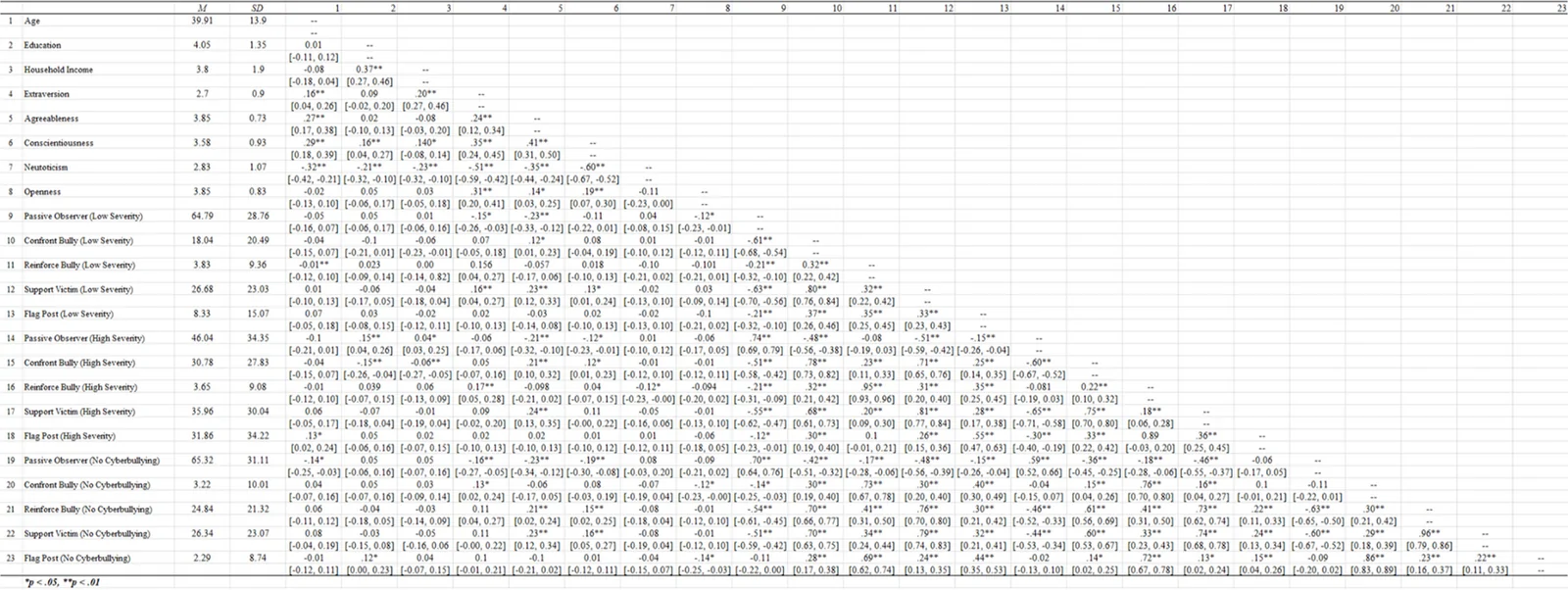
Mean, *SD,* and bivariate correlations for key study variables

### Extroversion

As shown in Tables 3 and 4, the results of the linear mixed model indicated that there was a nonsignificant three-way interaction between cyberbullying severity, cyberbystander behavior, and extroversion, *F*(8, 1176.97) = 0.47, *p* = 0.877. There were significant main effects of severity, *F*(2, 2031.40) = 22.02, *p* < 0.001, and type of cyberbystander behavior, *F*(4, 1152.64) = 515.55, *p* < 0.001, as well as a significant interaction between severity and behavior, *F*(8, 1176.97) = 115.67, *p* < 0.001. In addition, the interaction between cyberbystander behavior and extroversion was significant, *F*(4, 1152.64) = 6.91, *p* < 0.001.

**Table 3:** Linear mixed effects model with severity, bystander behavior, and extroversion

Effect	*df*	*F*	*p*
Severity	2, 2031.40	22.02	**< 0.001**
Bystander Behavior Type	4, 1152.64	515.55	**< 0.001**
Extroversion	1, 483.67	2.01	0.157
Severity x Bystander Behavior Type	8, 1176.97	115.67	**< 0.001**
Severity x Extroversion	2, 2031.40	0.04	0.964
Bystander Behavior Type x Extroversion	4, 1152.64	6.91	**< 0.001**
Severity x Bystander Behavior Type x Extroversion	8, 1176.97	0.47	0.877

Age, income, and education were included as covariates. Model estimated via REML with random intercepts per participant and a diagonal covariance structure across 15 repeated conditions. Degrees of freedom estimated using the Satterthwaite approximation. Type III sums of squares are used for fixed effects testing

Bold font indicates statistical significance

**Table 4:** Fixed-effect parameter estimates and associated standard error, test statistic, effect size, and *p*-value

Term	Estimate	*SE*	*df*	*t*	*p*
Intercept	5.77	2.44	295.53	2.36	**0.019**
High Severity	29.39	1.95	305.16	15.08	**< 0.001**
Low Severity	6.02	0.80	342.28	7.52	**< 0.001**
Passive Observer	62.90	1.91	305.15	32.95	**< 0.001**
Confront Bully	0.82	0.39	256.87	2.10	**0.037**
Reinforce Bully	18.07	1.13	321.77	15.99	**< 0.001**
Support Victim	23.43	1.24	317.47	18.90	**< 0.001**
Extroversion	1.36	0.61	374.90	2.23	**0.026**
High Severity x Passive Observer	−48.72	3.42	884.18	−14.23	**< 0.001**
High Severity x Confront Bully	−2.24	2.51	586.84	−0.89	0.372
High Severity x Reinforce Bully	−47.61	2.26	498.24	−21.06	**< 0.001**
High Severity x Support Victim	−19.83	2.84	788.12	−6.99	**< 0.001**
Low Severity x Passive Observer	−6.78	2.73	685.30	−2.48	**0.013**
Low Severity x Confront Bully	8.47	1.36	603.34	6.22	**< 0.001**
Low Severity x Reinforce Bully	−22.60	1.40	600.73	−16.20	**< 0.001**
Low Severity x Support Victim	−5.56	1.89	774.92	−2.94	**0.003**
High Severity x Extroversion	−0.42	1.95	305.16	−0.22	0.830
Low Severity x Extroversion	−0.62	0.80	342.28	−0.78	0.439
Passive Observer x Extroversion	−5.99	1.91	305.15	−3.13	**0.002**
Confront Bully x Extroversion	0.35	0.39	256.87	0.89	0.375
Reinforce Bully x Extroversion	2.11	1.13	321.77	1.86	0.064
Support Victim x Extroversion	1.33	1.24	317.47	1.07	0.285
High Severity x Passive Observer x Extroversion	3.31	3.43	884.18	0.97	0.334
High Severity x Confront Bully x Extroversion	0.27	2.51	586.84	0.11	0.915
High Severity x Reinforce Bully x Extroversion	−1.46	2.27	498.24	−0.64	0.520
High Severity x Support Victim x Extroversion	0.55	2.84	788.12	0.20	0.845
Low Severity x Passive Observer x Extroversion	1.27	2.74	685.30	0.47	0.642
Low Severity x Confront Bully x Extroversion	0.50	1.37	603.34	0.37	0.712
Low Severity x Reinforce Bully x Extroversion	−0.95	1.40	600.73	−0.68	0.495
Low Severity x Support Victim x Extroversion	1.68	1.89	774.92	0.89	0.374

The intercept corresponds to the unweighted grand mean for ratings. For each factor with *k* levels, *k* – 1 parameters are estimated. Age, income, and education were included as covariates but not shown. Degrees of freedom estimated using the Satterthwaite approximation

Bold font indicates statistical significance

Follow-up pairwise comparisons were performed to probe the interaction between cyberbullying severity and cyberbystander behavior (see Table 5). These results indicated that for screenshots depicting high severity cyberbullying, participants reported a greater likelihood of remaining a passive observer than all other cyberbystander behaviors—i.e., confronting the bully (*p* < 0.001), reinforcing the bully (*p* < 0.001), flagging the post (*p* < 0.001), or supporting the victim (*p* < 0.01). They were also more likely to confront the bully (*p* < 0.001), support the victim (*p* < 0.001), or flag the post (*p* < 0.001) than to reinforce the bully. For screenshots depicting low severity cyberbullying, participants also reported a greater likelihood of remaining a passive observer compared to all other behaviors (*p* < 0.001). Participants reported a greater likelihood of confronting the bully than reinforcing the bully or flagging the post (*p* < 0.001), but were more likely to support the victim than to confront the bully, reinforce the bully, or flag the post (*p* < 0.001). Lastly, participants also reported a greater likelihood of flagging the post than reinforcing the bully (*p* < 0.001). Although less interpretable, for screenshots that depicted no cyberbullying, participants reported a greater likelihood of remaining a passive observer than all other types of behavior (*p* < 0.001), a greater likelihood of reinforcing User B than confronting User B or flagging the post (*p* < 0.001), and a greater likelihood of supporting User A than confronting User B (*p* < 0.001), reinforcing User B’s behavior (*p* = 0.011) or flagging the post (*p* < 0.001).

**Table 5:** Pairwise comparisons of bystander behavior types by severity level for extroversion

Comparison	Mean Difference	*SE*	*z*	*p*^*a*^
High Severity				
Passive Observer vs Confront Bully	15.61	2.60	6.01	**< .001**
Passive Observer vs Reinforce Bully	4.73	2.11	20.71	< 0.001
Passive Observer vs Support Victim	10.59	2.67	3.97	**< 0.001**
Passive Observer vs Flag Post	14.19	2.84	4.99	**< 0.001**
Confront Bully vs Reinforce Bully	28.12	1.59	17.74	**< 0.001**
Confront Bully vs Support Victim	-5.02	2.28	-2.20	**0.279**
Confront Bully vs Flag Post	-1.42	2.48	-0.57	1.00
Reinforce Bully vs Support Victim	-33.14	1.70	-19.51	< 0.001
Reinforce Bully vs Flag Post	-29.55	1.96	-15.08	**< 0.001**
Support Victim vs Flag Post	3.59	2.55	1.41	**1.00**
Low Severity				
Passive Observer vs Confront Bully	46.84	2.10	22.36	**< 0.001**
Passive Observer vs Reinforce Bully	60.67	1.83	33.12	**< 0.001**
Passive Observer vs Support Victim	38.27	2.17	17.62	**< 0.001**
Passive Observer vs Flag Post	56.13	1.96	28.70	**< 0.001**
Confront Bully vs Reinforce Bully	13.83	1.11	12.45	**< 0.001**
Confront Bully vs Support Victim	−8.57	1.61	−5.32	**< 0.001**
Confront Bully vs Flag Post	9.29	1.31	7.11	**< 0.001**
Reinforce Bully vs Support Victim	−22.40	1.25	−17.92	**< 0.001**
Reinforce Bully vs Flag Post	−4.54	0.82	−5.54	**< 0.001**
Support Victim vs Flag Post	17.86	1.43	12.53	**< 0.001**
No Cyberbullying				
Passive Observer vs Confront Bully	62.10	1.91	32.48	**< 0.001**
Passive Observer vs Reinforce Bully	44.85	2.19	20.52	**< 0.001**
Passive Observer vs Support Victim	39.49	2.25	17.59	**< 0.001**
Passive Observer vs Flag Post	62.92	1.90	32.96	**< 0.001**
Confront Bully vs Reinforce Bully	−17.25	1.14	−15.19	**< 0.001**
Confront Bully vs Support Victim	−22.61	1.24	−18.17	**< 0.001**
Confront Bully vs Flag Post	0.82	0.39	2.09	0.373
Reinforce Bully vs Support Victim	−5.36	1.63	−3.28	**0.011**
Reinforce Bully vs Flag Post	18.06	1.13	15.98	**< 0.001**
Support Victim vs Flag Post	23.42	1.24	19.91	**< 0.001**

Bold font indicates statistical significance

^a^The *p*-values are adjusted using Bonferroni adjustment

Follow-up pairwise comparisons were also conducted to probe the interaction between cyberbystander behavior and extroversion at one standard deviation below the mean, the mean, and one standard deviation above the mean of extroversion (see Table 6). Across all levels of extroversion, participants reported a greater likelihood of remaining a passive observer than all other behaviors (*p* < 0.001). They were more likely to support the victim than to confront the bully, reinforce the bully, or flag the post (*p* < 0.001); were more likely to confront the bully than to reinforce the bully (*p* < 0.001); and were more likely to flag the post compared to reinforcing the bully (*p* < 0.01). Crucially, however, participants who were at the mean for extroversion reported a greater likelihood of confronting the bully than flagging the post (*p* = 0.022).

**Table 6:** Pairwise comparisons of bystander behavior types by severity level for extroversion

Comparison	Mean Difference	*SE*	*z*	*p*^*a*^
Low Extroversion (−1 *SD*)				
Passive Observer vs Confront Bully	46.57	1.81	25.68	**< 0.001**
Passive Observer vs Reinforce Bully	55.50	1.67	33.17	**< 0.001**
Passive Observer vs Support Victim	35.97	1.94	18.57	**< 0.001**
Passive Observer vs Flag Post	48.86	1.86	26.30	**< 0.001**
Confront Bully vs Reinforce Bully	8.93	1.06	8.45	**< 0.001**
Confront Bully vs Support Victim	−10.60	1.44	−7.36	**< 0.001**
Confront Bully vs Flag Post	2.29	1.33	1.72	0.857
Reinforce Bully vs Support Victim	−19.53	1.26	−15.52	**< 0.001**
Reinforce Bully vs Flag Post	−6.64	1.13	−5.86	**< 0.001**
Support Victim vs Flag Post	12.89	1.50	8.62	**< 0.001**
Average Extroversion				
Passive Observer vs Confront Bully	41.50	1.28	32.37	**< 0.001**
Passive Observer vs Reinforce Bully	49.74	1.18	42.04	**< 0.001**
Passive Observer vs Support Victim	29.44	1.37	21.49	**< 0.001**
Passive Observer vs Flag Post	44.40	1.32	33.76	**< 0.001**
Confront Bully vs Reinforce Bully	8.23	0.75	11.01	**< 0.001**
Confront Bully vs Support Victim	−12.07	1.02	−11.86	**< 0.001**
Confront Bully vs Flag Post	2.90	0.94	3.07	**0.022**
Reinforce Bully vs Support Victim	−8.23	0.75	−11.01	**< 0.001**
Reinforce Bully vs Flag Post	−20.30	0.89	−22.84	**< 0.001**
Support Victim vs Flag Post	−5.34	0.80	−6.66	**< 0.001**
High Extroversion (+1 *SD*)				
Passive Observer vs Confront Bully	36.44	1.82	20.06	**< 0.001**
Passive Observer vs Reinforce Bully	43.98	1.68	26.24	**< 0.001**
Passive Observer vs Support Victim	22.90	1.94	11.80	**< 0.001**
Passive Observer vs Flag Post	39.94	1.86	21.44	**< 0.001**
Confront Bully vs Reinforce Bully	7.54	1.06	7.11	**< 0.001**
Confront Bully vs Support Victim	−13.54	1.44	−9.39	**< 0.001**
Confront Bully vs Flag Post	3.50	1.34	2.62	0.089
Reinforce Bully vs Support Victim	−21.08	1.26	−16.73	**< 0.001**
Reinforce Bully vs Flag Post	−4.04	1.14	−3.55	**0.004**
Support Victim vs Flag Post	17.04	1.50	11.37	**< 0.001**

Bold font indicates statistical significance

^a^Denotes *p*-values using Bonferroni adjustment

In sum, in response to high severity cyberbullying, participants were most likely to remain a passive observer but when choosing to take action, they were most likely to support the victim and least likely to reinforce the bully. In response to low severity cyberbullying, participants were also most likely to remain a passive observer and more likely to support the victim than to engage in other cyberbystander behaviors. Although the likelihood of various cyberbystander behaviors was largely consistent across levels of extroversion, those who were moderately extroverted (within the sample) were more likely to confront the bully—a form of direct intervention—than they were to flag the post—an indirect behavior.

### Agreeableness

As shown in Tables 7 and 8, the results of the linear mixed model indicated a significant three-way interaction between cyberbullying severity, cyberbystander behavior, and agreeableness, *F*(8, 1175.99) = 4.94, *p* < 0.001. There were significant main effects of severity, *F*(2, 1997.35) = 23.16, *p* < 0.001, and cyberbystander behavior, *F*(4, 1141.02) = 531.01, *p* < 0.001, as well as a significant interaction between severity and behavior, *F*(8, 1175.99) = 120.77, *p* < 0.001, and between behavior and agreeableness, *F*(4, 1141.02) = 24.85, *p* < 0.001 (Table 9).

**Table 7:** Linear mixed effects model with severity, bystander behavior, and agreeableness

Effect	*df*	*F*	*p*
Severity	2, 1997.35	23.16	**< 0.001**
Bystander Behavior Type	4, 1141.02	531.01	**< 0.001**
Agreeableness	1, 484.18	0.009	0.922
Severity x Bystander Behavior Type	8, 1175.99	120.77	**< 0.001**
Severity x Agreeableness	2, 1997.35	1.07	0.345
Bystander Behavior Type x Agreeableness	4, 1141.02	24.85	**< 0.001**
Severity x Bystander Behavior Type x Agreeableness	8, 1175.99	4.94	**< 0.001**

Participant age, income, and education are treated as covariates. Degrees of freedom were calculated using the Satterthwaite method. Participant ID was treated as a random effects grouping factor. Type III sums of squares testing

**Table 8:** Fixed-effect parameter estimates and associated standard error, test statistic, effect size, and *p*-value

Term	Estimate	*SE*	*df*	*t*	*p*
Intercept	3.42	2.38	295.38	1.44	0.151
High Severity	29.40	1.95	305.08	15.12	**< 0.001**
Low Severity	6.10	0.80	341.47	7.60	**< 0.001**
Passive Observer	63.09	1.90	305.08	33.15	**< 0.001**
Confront Bully	0.82	0.39	259.37	2.11	**0.036**
Reinforce Bully	18.22	1.12	322.09	16.32	**< 0.001**
Support Victim	23.60	1.19	318.94	19.79	**< 0.001**
Agreeableness	−1.62	0.60	373.88	−2.71	**0.007**
High Severity x Passive Observer	−48.68	3.40	885.26	−14.30	**< 0.001**
High Severity x Confront Bully	−2.04	2.47	580.03	−0.83	0.410
High Severity x Reinforce Bully	−47.78	2.25	494.59	−21.23	**< 0.001**
High Severity x Support Victim	−19.75	2.78	774.03	−7.12	**< 0.001**
Low Severity x Passive Observer	−6.90	2.73	686.11	−2.53	**0.012**
Low Severity x Confront Bully	8.55	1.35	611.36	6.34	**< 0.001**
Low Severity x Reinforce Bully	−22.82	1.39	607.38	−16.47	**< 0.001**
Low Severity x Support Victim	−5.66	1.83	786.58	−3.09	**0.002**
High Severity x Agreeableness	1.93	1.95	305.08	0.99	0.323
Low Severity x Agreeableness	0.41	0.81	341.47	0.51	0.611
Passive Observer x Agreeableness	−5.15	1.91	305.08	−2.70	**0.007**
Confront Bully x Agreeableness	0.41	0.39	259.37	1.04	0.298
Reinforce Bully x Agreeableness	3.89	1.12	322.09	3.48	**< 0.001**
Support Victim x Agreeableness	6.45	1.20	318.94	5.39	**< 0.001**
High Severity x Passive Observer x Agreeableness	−2.39	3.42	885.26	−0.70	0.484
High Severity x Confront Bully x Agreeableness	4.77	2.48	580.03	1.92	0.055
High Severity x Reinforce Bully x Agreeableness	−6.11	2.26	494.59	−2.70	**0.007**
High Severity x Support Victim x Agreeableness	0.15	2.78	774.03	0.06	0.956
Low Severity x Passive Observer x Agreeableness	0.08	2.74	686.11	0.03	0.977
Low Severity x Confront Bully x Agreeableness	2.73	1.35	611.36	2.02	**0.151**
Low Severity x Reinforce Bully x Agreeableness	−3.93	1.39	607.38	−2.82	**< 0.001**
Low Severity x Support Victim x Agreeableness	−0.43	1.84	786.58	−0.24	0.814

The intercept corresponds to the unweighted grand mean for ratings. For each factor with *k* levels, *k* – 1 parameters are estimated. Age, income, and education were included as covariates but not shown. Degrees of freedom estimated using the Satterthwaite approximation

Bold font indicates statistical significance

**Table 9:** Three-way interaction between severity and bystander behavior at low, average, and high agreeableness

Comparison	*Mean Difference*	*SE*	*z*	*p* ^*a*^
High Severity * Low Agreeableness (−1 *SD*)				
Passive Observer vs Confront Bully	28.35	3.60	7.88	**< 0.001**
Passive Observer vs Reinforce Bully	49.30	2.95	16.74	**<.001**
Passive Observer vs Support Victim	24.71	3.69	6.70	**< 0.001**
Passive Observer vs Flag Post	21.95	3.98	5.51	**< 0.001**
Confront Bully vs Reinforce Bully	20.96	2.16	9.68	**< 0.001**
Confront Bully vs Support Victim	−3.64	3.10	−1.18	1.00
Confront Bully vs Flag Post	−6.40	3.45	−1.86	0.640
Reinforce Bully vs Support Victim	−24.60	2.31	−10.67	**< 0.001**
Reinforce Bully vs Flag Post	−27.35	2.76	−9.92	**< 0.001**
Support Victim vs Flag Post	−2.76	3.54	−0.78	1.00
High Severity * Average Agreeableness				
Passive Observer vs Confront Bully	15.62	2.55	6.13	**< 0.001**
Passive Observer vs Reinforce Bully	43.97	2.09	21.06	**< 0.001**
Passive Observer vs Support Victim	10.56	2.61	4.04	**< 0.001**
Passive Observer vs Flag Post	14.40	2.82	5.10	**< 0.001**
Confront Bully vs Reinforce Bully	28.35	1.53	18.48	**< 0.001**
Confront Bully vs Support Victim	−5.07	2.19	−2.31	0.213
Confront Bully vs Flag Post	−1.22	2.44	−0.50	1.00
Reinforce Bully vs Support Victim	−33.41	1.63	−20.45	**< 0.001**
Reinforce Bully vs Flag Post	−29.56	1.95	−15.13	**< 0.001**
Support Victim vs Flag Post	3.85	2.51	1.54	1.00
High Severity * High Agreeableness (+1 *SD*)				
Passive Observer vs Confront Bully	2.90	3.63	0.80	1.00
Passive Observer vs Reinforce Bully	38.63	2.97	13.02	**< 0.001**
Passive Observer vs Support Victim	−3.60	3.71	−0.97	1.00
Passive Observer vs Flag Post	6.86	4.01	1.71	0.881
Confront Bully vs Reinforce Bully	35.74	2.18	16.39	**< 0.001**
Confront Bully vs Support Victim	−6.49	3.12	−2.08	0.379
Confront Bully vs Flag Post	3.96	3.47	1.14	1.00
Reinforce Bully vs Support Victim	−42.23	2.32	−18.17	**< 0.001**
Reinforce Bully vs Flag Post	−31.78	2.78	−11.44	**< 0.001**
Support Victim vs Flag Post	10.45	3.56	2.93	**0.04**
Low Severity * Low Agreeableness (−1 *SD*)				
Passive Observer vs Confront Bully	55.03	2.94	18.73	**< 0.001**
Passive Observer vs Reinforce Bully	65.83	2.58	25.50	**< 0.001**
Passive Observer vs Support Victim	49.34	3.03	16.29	**< 0.001**
Passive Observer vs Flag Post	61.26	2.76	22.22	**< 0.001**
Confront Bully vs Reinforce Bully	10.80	1.54	7.01	**< 0.001**
Confront Bully vs Support Victim	−5.69	2.21	−2.57	0.103
Confront Bully vs Flag Post	6.23	1.82	3.42	**0.007**
Reinforce Bully vs Support Victim	−16.49	1.71	−9.66	**< 0.001**
Reinforce Bully vs Flag Post	−4.57	1.16	−3.94	**< 0.001**
Support Victim vs Flag Post	11.92	1.96	6.07	**< 0.001**
Low Severity * Average Agreeableness				
Passive Observer vs Confront Bully	46.81	2.08	22.48	**< 0.001**
Passive Observer vs Reinforce Bully	60.78	1.83	33.23	**< 0.001**
Passive Observer vs Support Victim	38.25	2.15	17.82	**< 0.001**
Passive Observer vs Flag Post	56.18	1.95	28.75	**< 0.001**
Confront Bully vs Reinforce Bully	13.97	1.09	12.79	**< 0.001**
Confront Bully vs Support Victim	−8.57	1.57	−5.47	**< 0.001**
Confront Bully vs Flag Post	9.37	1.29	7.26	**< 0.001**
Reinforce Bully vs Support Victim	−22.53	1.21	−18.62	**< 0.001**
Reinforce Bully vs Flag Post	−4.60	0.82	−5.60	**< 0.001**
Support Victim vs Flag Post	17.94	1.39	12.89	**< 0.001**
Low Severity * High Agreeableness (+1 *SD*)				
Passive Observer vs Confront Bully	38.60	2.96	13.04	**< 0.001**
Passive Observer vs Reinforce Bully	55.74	2.60	21.44	**< 0.001**
Passive Observer vs Support Victim	27.16	3.05	8.90	**< 0.001**
Passive Observer vs Flag Post	51.11	2.78	18.40	**< 0.001**
Confront Bully vs Reinforce Bully	17.14	1.55	11.04	**< 0.001**
Confront Bully vs Support Victim	−11.44	2.23	−5.14	**< 0.001**
Confront Bully vs Flag Post	12.51	1.84	6.82	**< 0.001**
Reinforce Bully vs Support Victim	−28.58	1.72	−16.62	**< 0.001**
Reinforce Bully vs Flag Post	−4.63	1.17	−3.96	**< 0.001**
Support Victim vs Flag Post	23.95	1.98	12.11	**< 0.001**
No Cyberbullying * Low Agreeableness (−1 *SD*)				
Passive Observer vs Confront Bully	67.82	2.69	25.21	**< 0.001**
Passive Observer vs Reinforce Bully	53.92	3.07	17.57	**< 0.001**
Passive Observer vs Support Victim	51.09	3.13	16.35	**< 0.001**
Passive Observer vs Flag Post	68.24	2.69	25.41	**< 0.001**
Confront Bully vs Reinforce Bully	−13.91	1.58	−8.79	**< 0.001**
Confront Bully vs Support Victim	−16.73	1.69	−9.90	**< 0.001**
Confront Bully vs Flag Post	0.42	0.55	0.75	1.00
Reinforce Bully vs Support Victim	−2.83	2.24	−1.26	1.00
Reinforce Bully vs Flag Post	14.32	1.58	9.09	**< 0.001**
Support Victim vs Flag Post	17.15	1.68	10.19	**< 0.001**
No Cyberbullying * Average Agreeableness				
Passive Observer vs Confront Bully	62.26	1.91	32.67	**< 0.001**
Passive Observer vs Reinforce Bully	44.87	2.17	20.64	**< 0.001**
Passive Observer vs Support Victim	39.49	2.21	17.83	**< 0.001**
Passive Observer vs Flag Post	63.09	1.90	33.15	**< 0.001**
Confront Bully vs Reinforce Bully	−17.40	1.12	−15.50	**< 0.001**
Confront Bully vs Support Victim	−22.78	1.20	−19.01	**< 0.001**
Confront Bully vs Flag Post	0.82	0.39	2.11	0.358
Reinforce Bully vs Support Victim	−5.38	1.59	−3.38	**0.008**
Reinforce Bully vs Flag Post	18.22	1.12	16.32	**< 0.001**
Support Victim vs Flag Post	23.60	1.19	19.80	**< 0.001**
No Cyberbullying * High Agreeableness (+1 *SD*)				
Passive Observer vs Confront Bully	56.70	2.71	20.92	**< 0.001**
Passive Observer vs Reinforce Bully	35.82	3.09	11.59	**< 0.001**
Passive Observer vs Support Victim	27.88	3.15	8.86	**< 0.001**
Passive Observer vs Flag Post	57.93	2.71	21.41	**< 0.001**
Confront Bully vs Reinforce Bully	−20.88	1.60	−13.09	**< 0.001**
Confront Bully vs Support Victim	−28.82	1.70	−16.92	**< 0.001**
Confront Bully vs Flag Post	1.23	0.55	2.22	.273
Reinforce Bully vs Support Victim	−7.94	2.26	−3.51	**.005**
Reinforce Bully vs Flag Post	22.11	1.59	13.93	**< 0.001**
Support Victim vs Flag Post	30.05	1.70	17.73	**< 0.001**

Bold font indicates statistical significance

^a^Denotes *p*-values using Bonferroni adjustment

To probe the three-way interaction, we examined the simple effects between cyberbullying severity and cyberbystander behavior at low (−1 *SD*), average, and high (+ 1 *SD*) levels of agreeableness. For screenshots depicting high severity cyberbullying, across levels of agreeableness, participants reported a greater willingness to confront the bully compared to reinforcing the bully (*p* < 0.001), as well as a greater willingness to support the victim and flag the behavior as opposed to reinforcing the bully (*p* < 0.001). However, participants with relatively lower or average levels of agreeableness reported a greater likelihood of remaining a passive observer compared to all other cyberbystander behaviors (*p* < 0.001). Participants who were high in agreeableness reported a greater likelihood of being a passive observer compared to reinforcing the bully (*p* < 0.001) and were also more likely to support the victim than flag the post (*p* = 0.04). In response to low severity cyberbullying, participants across all levels of agreeableness were more likely to remain a passive observer compared to all other behaviors (*p* < 0.001); were more likely to confront the bully than reinforce the bully (*p* < 0.001) or flag the post (*p* = 0.01); were more likely to flag the post compared to reinforcing the bully (*p* < 0.001); and were more likely to support the victim than to reinforce the bully (*p* < 0.01) or flag the post (*p* < 0.001). However, individuals who were average or high in agreeableness were also more likely to support the victim than confront the bully (*p* < 0.001).

Although less interpretable, after viewing screenshots depicting no cyberbullying, participants across all levels of agreeableness were more likely to remain a passive observer compared to all other behaviors (*p* < 0.001); were more likely to reinforce User B compared to confronting them or flagging the content (*p* < 0.001); and were more likely to support User A compared to confronting User B or flagging the post (*p* < 0.001). Notably, individuals who were average or high in agreeableness reported a greater likelihood of supporting the victim than reinforcing the bully (*p* = 0.01).

**Fig. 4:**
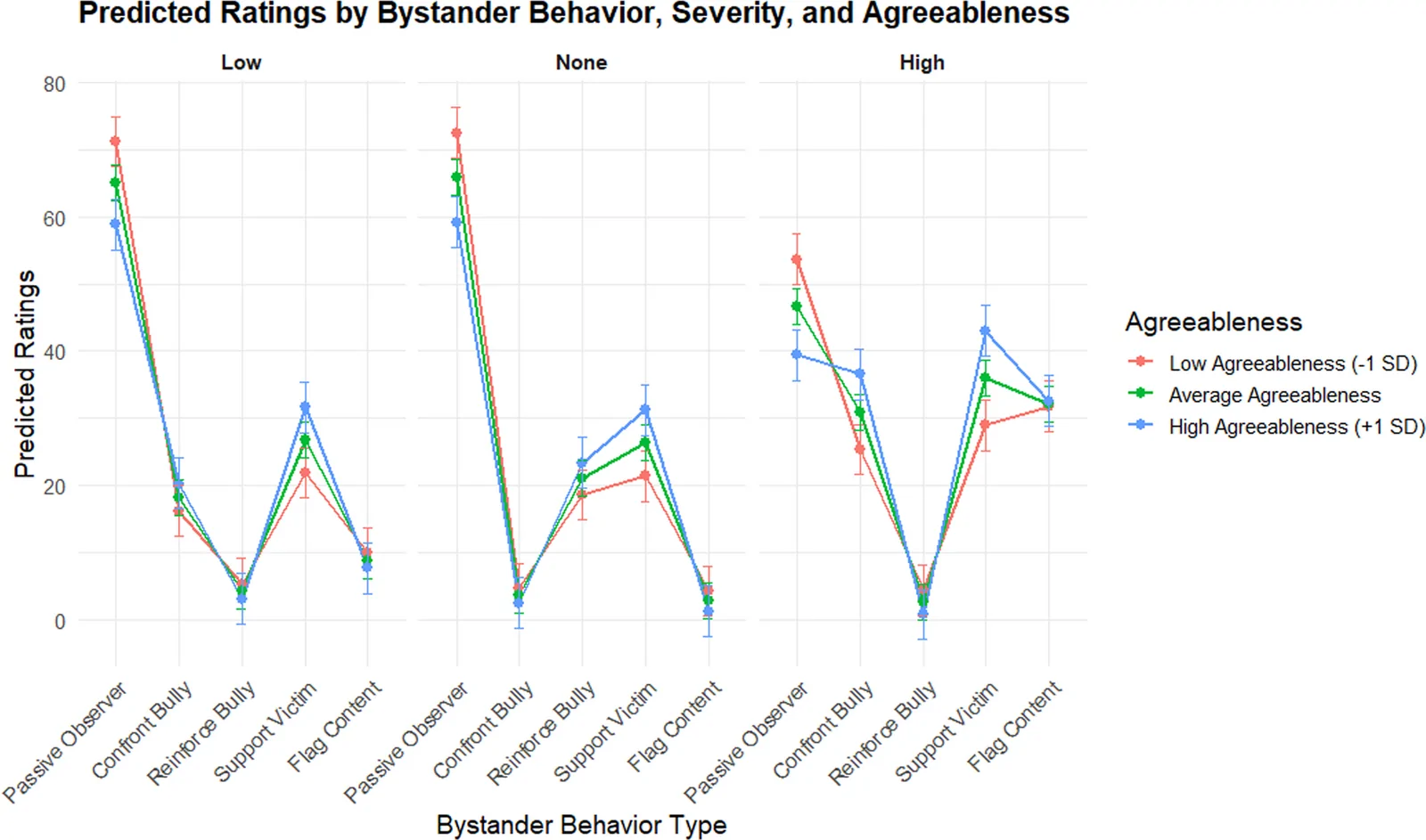
Predicted ratings by bystander behavior, severity, and agreeableness

In sum, as shown in Fig. 4, a significant three-way interaction emerged that seemed to be driven largely by differences in the likelihood of specific cyberbystander behaviors among participants who were relatively higher in agreeableness in response to high severity instances of cyberbullying. That is, those higher in agreeableness reported a greater likelihood of passively observing, and a higher likelihood of confronting the bully *or* supporting the victim than of reinforcing the bully, whereas participants who were lower or average in agreeableness reported a greater likelihood of remaining a passive observer than all other cyberbystander behaviors. Behaviors in response to low severity cyberbullying were more uniform across all levels of agreeableness, highlighting the potential role of agreeableness in uniquely predicting bystander action in more severe bullying instances.

### Conscientiousness

As shown in Tables 10 and 11, in the linear mixed model for conscientiousness, there was a nonsignificant three-way interaction between cyberbullying severity, cyberbystander behavior, and conscientiousness, *F*(8, 1161.39) = 1.15, *p* = 0.327, significant main effects of severity, *F*(2, 2007.33) = 22.17, *p* < 0.001, and cyberbystander behavior, *F*(4, 1144.74) = 523.02, *p* < 0.001, and significant interactions between severity and behavior, *F*(8, 1161.39) = 115.55, *p* < 0.001, and between behavior and conscientiousness, *F*(4, 1144.74) = 16.91, *p* < 0.001.

**Table 10:** Linear mixed effects model with severity, bystander behavior, and consciousness

Effect	*df*	*F*	*p*
Severity	2, 2007.33	22.17	**< 0.001**
Bystander Behavior Type	4, 1144.74	523.02	**< 0.001**
Conscientiousness	1, 460.00	0.45	0.502
Severity x Bystander Behavior Type	8, 1161.39	115.55	**< 0.001**
Severity x Conscientiousness	2, 2007.33	0.08	0.926
Bystander Behavior Type x Conscientiousness	4, 1144.74	16.91	**< 0.001**
Severity x Bystander Behavior Type x Conscientiousness	8, 1161.39	1.15	.327

Age, income, and education were included as covariates. Model estimated via REML with random intercepts per participant and a diagonal covariance structure across 15 repeated conditions. Degrees of freedom estimated using the Satterthwaite approximation. Type III sums of squares are used for fixed effects testing

Bold font indicates statistical significance

**Table 11:** Fixed-effect parameter estimates and associated standard error, test statistic, effect size, and* p*-value

Term	Estimate	*SE*	*df*	*t*	*p*
Intercept	4.95	2.61	292.98	1.90	**0.058**
High Severity	29.30	1.96	302.95	14.95	**< 0.001**
Low Severity	6.02	0.80	339.53	7.50	**< 0.001**
Passive Observer	63.45	1.91	303.06	33.28	**< 0.001**
Confront Bully	0.83	0.39	251.02	2.13	**0.034**
Reinforce Bully	17.94	1.12	319.76	15.99	**< 0.001**
Support Victim	23.35	1.23	315.44	18.97	**< 0.001**
Conscientiousness	−0.13	0.64	365.15	−0.20	0.841
High Severity x Passive Observer	−48.76	3.43	878.18	−14.22	**<0 .001**
High Severity x Confront Bully	−2.10	2.51	580.76	−0.84	0.404
High Severity x Reinforce Bully	−47.40	2.27	491.04	−20.90	**< 0.001**
High Severity x Support Victim	−19.61	2.84	778.26	−6.91	**< 0.001**
Low Severity x Passive Observer	−6.96	2.75	680.51	−2.53	**0.012**
Low Severity x Confront Bully	8.43	1.37	598.67	6.18	**< 0.001**
Low Severity x Reinforce Bully	−22.47	1.39	600.87	−16.16	**< 0.001**
Low Severity x Support Victim	−5.43	1.89	769.50	−2.88	**0.004**
High Severity x Conscientiousness	0.70	1.97	302.95	0.35	0.725
Low Severity x Conscientiousness	0.33	0.81	339.53	0.41	0.685
Passive Observer x Conscientiousness	−4.88	1.92	303.06	−2.55	**0.011**
Confront Bully x Conscientiousness	0.88	0.39	251.02	2.25	**0.026**
Reinforce Bully x Conscientiousness	2.79	1.13	319.76	2.47	**0.014**
Support Victim x Conscientiousness	3.49	1.24	315.44	2.82	**0.005**
High Severity x Passive Observer x Conscientiousness	0.63	3.45	878.18	0.18	0.855
High Severity x Confront Bully x Conscientiousness	1.79	2.53	580.76	0.71	0.480
High Severity x Reinforce Bully x Conscientiousness	−3.30	2.28	491.04	−1.45	.0149
High Severity x Support Victim x Conscientiousness	−0.90	2.86	778.26	−0.32	0.753
Low Severity x Passive Observer x Conscientiousness	2.08	2.76	680.51	0.75	0.452
Low Severity x Confront Bully x Conscientiousness	0.26	1.37	598.67	0.19	0.851
Low Severity x Reinforce Bully x Conscientiousness	−2.89	1.40	600.87	−2.07	**0.039**
Low Severity x Support Victim x Conscientiousness	−0.89	1.90	769.50	−0.47	0.641

The intercept corresponds to the unweighted grand mean for ratings. For each factor with* k* levels, *k* – 1 parameters are estimated. Age, income, and education were included as covariates but not shown. Degrees of freedom estimated using the Satterthwaite approximation

Bold font indicates statistical significance

Pairwise comparisons probing the interaction between cyberbullying severity and cyberbystander behavior indicated that in response to high severity cyberbullying, participants were more likely to remain a passive observer compared to all other cyberbystander behaviors (*p* < 0.001); were more likely to confront the bully (*p* < 0.001) or flag the post (*p* < 0.001) than to reinforce the bully; and were more likely to support the victim than to reinforce the bully (*p* < 0.001) (see Table 12). In response to low severity cyberbullying, participants reported a greater likelihood of remaining a passive observer than all other cyberbystander behaviors (*p* < 0.001); were more likely to confront the bully or flag the post than reinforce the bully (*p* < 0.001); were more likely to confront the bully than flag the post (*p* < 0.001); and were more likely to support the victim than to confront the bully, reinforce the bully, or flag the post (*p* < 0.001). In response to screenshots depicting no cyberbullying, participants reported a greater likelihood of remaining a passive observer than all other types of behavior (*p* < 0.001), were more likely to reinforce User B than confront User B or flag the post (*p* < 0.001), and were more likely to support User A than to confront User B (*p* < 0.001), reinforce the bully (*p* < 0.01), or flag the post (*p* < 0.001).

**Table 12:** Pairwise comparisons of bystander behavior types by severity level for conscientiousness

Comparison	Mean Difference	*SE*	*z*	*p*^*a*^
High Severity				
Passive Observer vs Confront Bully	16.03	2.60	6.17	**< 0.001**
Passive Observer vs Reinforce Bully	44.18	2.11	20.91	**< 0.001**
Passive Observer vs Support Victim	11.01	2.67	4.13	**< 0.001**
Passive Observer vs Flag Post	14.73	2.85	5.17	**< 0.001**
Confront Bully vs Reinforce Bully	28.16	1.58	17.79	**< 0.001**
Confront Bully vs Support Victim	−5.01	2.27	−2.21	0.278
Confront Bully vs Flag Post	−1.30	2.48	−0.52	1.00
Reinforce Bully vs Support Victim	−33.17	1.70	−19.55	**< 0.001**
Reinforce Bully vs Flag Post	−29.45	1.97	−14.95	**< 0.001**
Support Victim vs Flag Post	3.72	2.56	1.45	1.00
Low Severity				
Passive Observer vs Confront Bully	47.27	2.12	22.34	**< 0.001**
Passive Observer vs Reinforce Bully	61.05	1.86	32.91	**< 0.001**
Passive Observer vs Support Victim	38.63	2.20	17.59	**< 0.001**
Passive Observer vs Flag Post	56.52	1.98	28.57	**< 0.001**
Confront Bully vs Reinforce Bully	13.79	1.11	12.39	**< 0.001**
Confront Bully vs Support Victim	−8.64	1.62	−5.34	**< 0.001**
Confront Bully vs Flag Post	9.25	1.31	7.07	**< 0.001**
Reinforce Bully vs Support Victim	−22.43	1.26	−17.83	**< 0.001**
Reinforce Bully vs Flag Post	−4.54	0.82	−5.52	**< 0.001**
Support Victim vs Flag Post	17.89	1.43	12.49	**< 0.001**
No Cyberbullying				
Passive Observer vs Confront Bully	62.68	1.91	32.83	**< 0.001**
Passive Observer vs Reinforce Bully	45.59	2.18	20.91	**< 0.001**
Passive Observer vs Support Victim	40.18	2.24	17.95	**< 0.001**
Passive Observer vs Flag Post	63.50	1.91	33.31	**< 0.001**
Confront Bully vs Reinforce Bully	−17.09	1.13	−15.16	**< 0.001**
Confront Bully vs Support Victim	−22.50	1.24	−18.22	**< 0.001**
Confront Bully vs Flag Post	0.82	0.39	2.10	0.363
Reinforce Bully vs Support Victim	−5.41	1.62	−3.33	**0.009**
Reinforce Bully vs Flag Post	17.91	1.12	15.96	**< 0.001**
Support Victim vs Flag Post	23.32	1.23	18.94	**< 0.001**

Bold font indicates statistical significance

^a^Denotes *p*-values using Bonferroni adjustment

Follow-up pairwise comparisons were also conducted to probe the interaction between cyberbystander behavior and conscientiousness at one standard deviation below the mean, at the mean, and at one standard deviation above the mean of conscientiousness (see Table 13). Across all levels of conscientiousness, participants reported a greater likelihood of remaining a passive observer than all other behaviors (*p* < 0.001); were more likely to confront the bully than to reinforce the bully (*p* < 0.001); were more likely to support the victim than to confront the bully, reinforce the bully, or flag the post (*p* < 0.001); and were more likely to flag the post than to reinforce the bully (*p* < 0.001). Participants with average or relatively higher levels of conscientiousness, however, reported a greater likelihood of confronting the bully than flagging the post (*p*s < 0.01).

**Table 13:** Pairwise comparisons of bystander behavior types by conscientiousness

Comparison	Mean Difference	*SE*	*z*	*p*^*a*^
Low Conscientiousness (- 1 *SD*)				
Passive Observer vs Confront Bully	47.47	1.81	26.17	**< 0.001**
Passive Observer vs Reinforce Bully	54.93	1.67	32.83	**< 0.001**
Passive Observer vs Support Victim	36.75	1.94	18.97	**< 0.001**
Passive Observer vs Flag Post	48.85	1.86	26.24	**< 0.001**
Confront Bully vs Reinforce Bully	7.45	1.05	7.08	**< 0.001**
Confront Bully vs Support Victim	−10.73	1.44	−7.47	**< 0.001**
Confront Bully vs Flag Post	1.38	1.33	1.03	1.00
Reinforce Bully vs Support Victim	−18.18	1.25	−14.51	**< 0.001**
Reinforce Bully vs Flag Post	−6.08	1.13	−5.36	**< 0.001**
Support Victim vs Flag Post	12.10	1.50	8.09	**< 0.001**
Average Conscientiousness				
Passive Observer vs Confront Bully	41.94	1.29	32.64	**< 0.001**
Passive Observer vs Reinforce Bully	50.23	1.19	42.35	**< 0.001**
Passive Observer vs Support Victim	29.87	1.37	21.76	**< 0.001**
Passive Observer vs Flag Post	44.88	1.32	34.00	**< 0.001**
Confront Bully vs Reinforce Bully	8.29	0.75	11.12	**< 0.001**
Confront Bully vs Support Victim	−12.06	1.02	−11.86	**< 0.001**
Confront Bully vs Flag Post	2.94	0.95	3.11	**0.019**
Reinforce Bully vs Support Victim	−20.36	0.89	−22.92	**< 0.001**
Reinforce Bully vs Flag Post	−5.35	0.80	−6.66	**< 0.001**
Support Victim vs Flag Post	15.00	1.06	14.15	**< 0.001**
High Conscientiousness (+ 1 *SD*)				
Passive Observer vs Confront Bully	36.40	1.83	19.87	**< 0.001**
Passive Observer vs Reinforce Bully	45.53	1.69	26.94	**< 0.001**
Passive Observer vs Support Victim	23.00	1.96	11.76	**< 0.001**
Passive Observer vs Flag Post	40.90	1.88	21.74	**< 0.001**
Confront Bully vs Reinforce Bully	9.13	1.06	8.58	**< 0.001**
Confront Bully vs Support Victim	−13.40	1.45	−9.24	**< 0.001**
Confront Bully vs Flag Post	4.50	1.35	3.34	**0.009**
Reinforce Bully vs Support Victim	−22.53	1.27	−17.80	**< 0.001**
Reinforce Bully vs Flag Post	−4.63	1.15	−4.04	**< 0.001**
Support Victim vs Flag Post	17.90	1.51	11.85	**< 0.001**

Bold font indicates statistical significance

^a^Denotes *p*-values using Bonferroni adjustment

### Neuroticism

A shown in Tables 14 and 15, the results of the linear mixed model revealed a nonsignificant three-way interaction between cyberbullying severity, cyberbystander behavior, and neuroticism, *F*(8, 1173.58) = 0.29, *p* = 0.971, significant main effects of severity, *F*(2, 2001.39) = 21.46, *p* < 0.001, and cyberbystander behavior, *F*(4, 1155.20) = 507.48, *p* < 0.001, and a significant interaction between severity and behavior, *F*(8, 1173.58) = 114.19, *p* < 0.001. There were no significant main effects of or two-way interactions with neuroticism (Table 16).

**Table 14:** Linear mixed effects model with severity, bystander behavior, and neuroticism

Effect	*df*	*F*	*p*
Severity	2, 2001.39	21.46	**< 0.001**
Bystander Behavior Type	4, 1155.20	507.48	**< 0.001**
Neuroticism	1, 455.93	0.67	0.413
Severity x Bystander Behavior Type	8, 1173.58	114.19	**< 0.001**
Severity x Neuroticism	2, 2001.39	0.14	0.873
Bystander Behavior Type x Neuroticism	4, 1155.20	0.92	0.454
Severity x Bystander Behavior Type x Neuroticism	8, 1173.58	0.29	0.971

Age, income, and education were included as covariates. Model estimated via REML with random intercepts per participant and a diagonal covariance structure across 15 repeated conditions. Degrees of freedom estimated using the Satterthwaite approximation. Type III sums of squares are used for fixed effects testing

Bold font indicates statistical significance

**Table 15:** Fixed-effect parameter estimates and associated standard error, test statistic, effect size, and *p*-value

Term	Estimate	*SE*	*df*	*t*	*p*
Intercept	5.86	2.64	293.92	2.22	**0.027**
High Severity	29.28	1.95	304.07	15.01	**< 0.001**
Low Severity	6.05	0.80	340.47	7.53	**< 0.001**
Passive Observer	63.01	1.94	303.81	32.53	**< 0.001**
Confront Bully	0.81	0.39	256.78	2.08	**0.039**
Reinforce Bully	18.08	1.14	320.20	15.89	**< 0.001**
Support Victim	23.25	1.24	316.27	18.75	**< 0.001**
Neuroticism	−0.59	0.65	363.77	−0.91	0.363
High Severity x Passive Observer	−48.56	3.45	881.51	−14.09	**< 0.001**
High Severity x Confront Bully	−2.32	2.50	582.93	−0.93	0.354
High Severity x Reinforce Bully	−47.51	2.27	498.42	−20.96	**< .001**
High Severity x Support Victim	−19.68	2.84	785.59	−6.94	**< .001**
Low Severity x Passive Observer	−6.75	2.77	680.93	−2.44	**0.015**
Low Severity x Confront Bully	8.36	1.36	601.85	6.13	**< .001**
Low Severity x Reinforce Bully	−22.63	1.40	596.59	−16.13	**< .001**
Low Severity x Support Victim	−5.38	1.90	771.95	−2.83	**0.005**
High Severity x Neuroticism	0.04	1.96	304.07	0.02	0.985
Low Severity x Neuroticism	0.03	0.81	340.47	0.03	0.976
Passive Observer x Neuroticism	2.42	1.95	303.81	1.25	0.214
Confront Bully x Neuroticism	−0.40	0.39	256.78	−1.03	0.306
Reinforce Bully x Neuroticism	−1.05	1.14	320.20	−0.92	0.360
Support Victim x Neuroticism	−1.24	1.25	316.27	−1.00	0.318
High Severity x Passive Observer x Neuroticism	−2.06	3.46	881.51	−0.59	0.553
High Severity x Confront Bully x Neuroticism	0.49	2.51	582.93	0.20	0.845
High Severity x Reinforce Bully x Neuroticism	0.86	2.28	498.42	0.38	0.707
High Severity x Support Victim x Neuroticism	0.19	2.85	785.59	0.07	0.948
Low Severity x Passive Observer x Neuroticism	−1.28	2.78	680.93	−0.46	0.645
Low Severity x Confront Bully x Neuroticism	1.12	1.37	601.85	0.82	0.416
Low Severity x Reinforce Bully x Neuroticism	0.43	1.41	596.59	0.30	0.762
Low Severity x Support Victim x Neuroticism	1.23	1.91	771.95	0.64	0.520

The intercept corresponds to the unweighted grand mean for ratings. For each factor with *k* levels, *k* – 1 parameters are estimated. Age, income, and education were included as covariates but not shown. Degrees of freedom estimated using the Satterthwaite approximation

Bold font indicates statistical significance

**Table 16:** Pairwise comparisons of bystander behavior types by severity level for neuroticism

Comparison	Mean Difference	*SE*	*z*	*p* ^*a*^
High Severity				
Passive Observer vs Confront Bully	15.96	2.60	6.14	**< 0.001**
Passive Observer vs Reinforce Bully	43.88	2.12	20.67	**< 0.001**
Passive Observer vs Support Victim	10.88	2.68	4.06	**< 0.001**
Passive Observer vs Flag Post	14.45	2.85	5.07	**< 0.001**
Confront Bully vs Reinforce Bully	27.93	1.57	17.74	**< 0.001**
Confront Bully vs Support Victim	−5.08	2.27	−2.24	0.256
Confront Bully vs Flag Post	−1.51	2.47	−0.61	1.00
Reinforce Bully vs Support Victim	−33.00	1.70	−19.44	**< 0.001**
Reinforce Bully vs Flag Post	−29.44	1.96	−15.01	**< 0.001**
Support Victim vs Flag Post	3.56	2.55	1.40	1.00
Low Severity				
Passive Observer vs Confront Bully	47.10	2.12	22.24	**< 0.001**
Passive Observer vs Reinforce Bully	60.82	1.86	32.74	**< 0.001**
Passive Observer vs Support Victim	38.40	2.20	17.44	**< 0.001**
Passive Observer vs Flag Post	56.27	1.98	28.39	**< 0.001**
Confront Bully vs Reinforce Bully	13.73	1.11	12.37	**< 0.001**
Confront Bully vs Support Victim	−8.69	1.62	−5.36	**< 0.001**
Confront Bully vs Flag Post	9.17	1.31	7.02	**< 0.001**
Reinforce Bully vs Support Victim	−22.42	1.26	−17.74	**< 0.001**
Reinforce Bully vs Flag Post	−4.56	0.82	−5.55	**< 0.001**
Support Victim vs Flag Post	17.87	1.44	12.41	**< 0.001**
No Cyberbullying				
Passive Observer vs Confront Bully	62.22	1.94	32.07	**< 0.001**
Passive Observer vs Reinforce Bully	44.96	2.22	20.30	**< 0.001**
Passive Observer vs Support Victim	39.79	2.27	17.54	**< 0.001**
Passive Observer vs Flag Post	63.03	1.94	32.54	**< 0.001**
Confront Bully vs Reinforce Bully	−17.27	1.14	−15.12	**< 0.001**
Confront Bully vs Support Victim	−22.43	1.24	−18.03	**< 0.001**
Confront Bully vs Flag Post	0.80	0.39	2.07	0.393
Reinforce Bully vs Support Victim	−5.17	1.64	−3.15	**0.017**
Reinforce Bully vs Flag Post	18.07	1.14	15.88	**< 0.001**
Support Victim vs Flag Post	23.24	1.24	18.74	**< 0.001**

Bold font indicates statistical significance

^a^Denotes *p*-values using Bonferroni adjustment

Pairwise comparisons probing the interaction between severity and behavior indicated that in response to high severity cyberbullying, participants once again reported a greater likelihood of remaining a passive observer than all other cyberbystander behaviors (*p* < 0.01); were more likely to confront the bully (*p* < 0.001) than to reinforce the bully; were less likely to reinforce the bully than flag the post (*p* < 0.001); and were more likely to support the victim than to reinforce the bully (*p* < 0.001). In response to low severity cyberbullying, participants were once again more likely to remain a passive observer than all other behaviors (*p* < 0.001); were more likely to confront the bully than to reinforce the bully or flag the post (*p* < 0.001); were less likely to reinforce the bully than to flag the post (*p* < 0.001); and were more likely to support the victim than to confront the bully, reinforce the bully, or flag the post (*p* < 0.001). In response to screenshots containing no cyberbullying, participants reported a greater likelihood of remaining a passive observer than all other types of behavior (*p* < 0.001), were more likely to reinforce User B than to confront User B or flag the post (*p* < 0.001), and were more likely to support User A than to confront User B (*p* < 0.001), reinforce User B (*p* = 0.017), or flag the post (*p* < 0.001).

### Openness

As shown in Tables 17 and 18, there was a nonsignificant three-way interaction between cyberbullying severity, cyberbystander behavior, and openness, *F*(8, 1175.92) = 0.41, *p* = 0.916, significant main effects of severity, *F*(2, 1996.36) = 22.41, *p* < 0.001, cyberbystander behavior, *F*(4, 1153.59) = 504.38, *p* < 0.001, and openness, *F*(1, 499.82) = 5.50, *p* = 0.019, and a significant interaction between severity and behavior, *F*(8, 1175.92) = 115.19, *p* < 0.001. The two-way interactions that included openness were nonsignificant.

**Table 17:** Linear mixed effects model with severity, bystander behavior, and openness

Effect	*df*	*F*	*p*
Severity	2, 1996.36	22.41	**< 0.001**
Bystander Behavior Type	4, 1153.59	504.38	**< 0.001**
Openness	1, 499.82	5.50	**0.019**
Severity x Bystander Behavior Type	8, 1175.92	115.19	**< 0.001**
Severity x Openness	2, 1996.36	0.03	0.969
Bystander Behavior Type x Openness	4, 1153.59	1.79	0.128
Severity x Bystander Behavior Type x Openness	8, 1175.92	0.41	0 .916

Age, income, and education were included as covariates. Model estimated via REML with random intercepts per participant and a diagonal covariance structure across 15 repeated conditions. Degrees of freedom estimated using the Satterthwaite approximation. Type III sums of squares used for fixed effects testing

Bold font indicates statistical significance

**Table 18:** Fixed-effect parameter estimates and associated standard error, test statistic, effect size, and *p*-value

Term	Estimate	*SE*	*df*	*t*	*p*
Intercept	4.04	2.40	294.93	1.69	0.093
High Severity	29.43	1.96	303.86	15.03	**< 0.001**
Low Severity	6.06	0.80	339.86	7.54	**< 0.001**
Passive Observer	62.80	1.95	303.60	32.24	**< 0.001**
Confront Bully	0.82	0.39	256.68	2.11	**0.036**
Reinforce Bully	18.12	1.14	319.64	15.90	**< 0.001**
Support Victim	23.58	1.24	315.74	18.96	**< 0.001**
Openness	−2.02	0.59	373.74	−3.43	**< 0.001**
High Severity x Passive Observer	−48.60	3.46	882.10	−14.06	**< 0.001**
High Severity x Confront Bully	−2.07	2.51	583.31	−0.82	0.412
High Severity x Reinforce Bully	−47.72	2.28	497.43	−20.98	**< 0.001**
High Severity x Support Victim	−19.83	2.85	785.00	−6.96	**< 0.001**
Low Severity x Passive Observer	−6.64	2.79	679.90	−2.39	**0.017**
Low Severity x Confront Bully	8.50	1.36	601.83	6.23	**< 0.001**
Low Severity x Reinforce Bully	−22.70	1.41	596.33	−16.14	**< 0.001**
Low Severity x Support Victim	−5.71	1.90	771.69	−3.01	**0.003**
High Severity x Openness	−0.47	1.96	303.86	−0.24	0.810
Low Severity x Openness	−0.17	0.80	339.86	−0.22	0.829
Passive Observer x Openness	−1.18	1.95	303.60	−0.61	0.546
Confront Bully x Openness	0.28	0.39	256.68	0.73	0.468
Reinforce Bully x Openness	1.47	1.14	319.64	1.29	0.199
Support Victim x Neuroticism	1.58	1.24	315.74	1.28	0.203
High Severity x Passive Observer x Openness	1.16	3.45	882.10	0.34	0.737
High Severity x Confront Bully x Openness	1.91	2.51	583.31	0.76	0.447
High Severity x Reinforce Bully x Openness	−0.17	2.27	497.43	−0.08	0.940
High Severity x Support Victim x Openness	0.51	2.85	785.00	0.18	0.858
Low Severity x Passive Observer x Openness	−0.18	2.78	679.90	−0.07	0.947
Low Severity x Confront Bully x Openness	1.46	1.36	601.83	1.07	0.284
Low Severity x Reinforce Bully x Openness	−0.86	1.40	596.33	−0.61	0.540
Low Severity x Support Victim x Openness	1.07	1.90	771.69	0.57	0.572

The intercept corresponds to the unweighted grand mean for ratings. For each factor with *k* levels, *k* – 1 parameters are estimated. Age, income, and education were included as covariates but not shown. Degrees of freedom estimated using the Satterthwaite approximation

Bold font indicates statistical significance

As shown in Table 19, pairwise comparisons revealed once again that with high severity cyberbullying, participants reported a greater likelihood of remaining a passive observer than all other behaviors (*p* < 0.01) and were more likely to confront the bully (*p* < 0.001), support the victim (*p* < 0.001), or flag the behavior (*p* < 0.01) than to reinforce the bully. With low severity cyberbullying, participants were more likely to remain a passive observer than all other behaviors (*p* < 0.001); were more likely to confront the bully than to reinforce the bully or flag the post (*p* < 0.001); were more likely to flag the post than reinforce the bully (*p* < 0.001); and were more likely to support the victim than to confront the bully, reinforce the bully, or flag the post (*p* < 0.001). In response to screenshots depicting no cyberbullying, participants were more likely to remain a passive observer than all other types of behavior (*p* < 0.001), were more likely to reinforce User B than to confront User B or flag the post (*p* < 0.001), and were more likely to support User A than to confront User B (*p* < 0.001), reinforce User B (*p* = 0.01), or flag the post (*p* < 0.001).

**Table 19:** Pairwise comparisons of bystander behavior types by severity level for openness

Comparison	Mean Difference	*SE*	*z*	*p*^*a*^
High Severity				
Passive Observer vs Confront Bully	15.45	2.61	5.92	**< 0.001**
Passive Observer vs Reinforce Bully	43.80	2.12	20.65	**< 0.001**
Passive Observer vs Support Victim	10.44	2.68	3.89	**0.001**
Passive Observer vs Flag Post	14.20	2.86	4.97	**< 0.001**
Confront Bully vs Reinforce Bully	28.35	1.59	17.89	**< 0.001**
Confront Bully vs Support Victim	−5.00	2.28	−2.19	0.288
Confront Bully vs Flag Post	−1.24	2.48	−0.50	1.00
Reinforce Bully vs Support Victim	−33.35	1.71	−19.54	**< 0.001**
Reinforce Bully vs Flag Post	−29.59	1.97	−15.04	**< 0.001**
Support Victim vs Flag Post	3.76	2.56	1.47	1.00
Low Severity				
Passive Observer vs Confront Bully	46.83	2.13	22.03	**< 0.001**
Passive Observer vs Reinforce Bully	60.72	1.87	32.52	**< 0.001**
Passive Observer vs Support Victim	38.27	2.21	17.34	**< 0.001**
Passive Observer vs Flag Post	56.15	1.99	28.22	**< 0.001**
Confront Bully vs Reinforce Bully	13.89	1.11	12.50	**< 0.001**
Confront Bully vs Support Victim	−8.56	1.62	−5.29	**< 0.001**
Confront Bully vs Flag Post	9.32	1.31	7.13	**< 0.001**
Reinforce Bully vs Support Victim	−22.45	1.26	−17.82	**< 0.001**
Reinforce Bully vs Flag Post	−4.57	0.82	−5.55	**< 0.001**
Support Victim vs Flag Post	17.88	1.44	12.45	**< 0.001**
No Cyberbullying				
Passive Observer vs Confront Bully	61.98	1.95	31.77	**< 0.001**
Passive Observer vs Reinforce Bully	44.67	2.23	20.07	**< 0.001**
Passive Observer vs Support Victim	39.21	2.28	17.19	**< 0.001**
Passive Observer vs Flag Post	62.80	1.95	32.24	**< 0.001**
Confront Bully vs Reinforce Bully	−17.31	1.15	−15.11	**< 0.001**
Confront Bully vs Support Victim	−22.76	1.25	−18.24	**< 0.001**
Confront Bully vs Flag Post	0.82	0.39	2.11	0.357
Reinforce Bully vs Support Victim	−5.46	1.65	−3.32	**0.010**
Reinforce Bully vs Flag Post	18.13	1.14	15.90	**< 0.001**
Support Victim vs Flag Post	23.59	1.24	18.96	**< 0.001**

Bold font indicates statistical significance

^a^Denotes *p*-values using Bonferroni adjustment

## Discussion

The present study sought to contribute to the emerging literature on cyberbystanders by investigating the extent to which the Big Five personality traits predict different cyberbystander behaviors—including remaining a passive observer, intervening to either reinforce a bully, confront a bully, or support a victim, and flagging or reporting cyberbullying content—and whether these associations vary depending on the severity of the cyberbullying instance. Specifically, participants viewed screenshots depicting ostensible social media interactions determined (via pilot testing) to capture three distinct cyberbullying severity levels (no cyberbullying, low severity, and high severity), reported their likelihood of engaging in a range of cyberbystander behaviors in response to each interaction, and completed a measure of the Big Five traits.

A series of linear mixed effects models yielded several notable insights. Across all cyberbullying severity levels and Big Five traits, participants exhibited a consistent behavioral preference: the likelihood of remaining a passive observer exceeded that of all other types of behaviors, while supporting the victim was more likely than flagging the post, confronting the bully, or reinforcing the bully. That is, mirroring previous research [68, 69], the participants in our study demonstrated a reluctance to intervene—directly or indirectly—into a social media interaction between other users. Broadly, our results also showed a general trend toward being more likely to support a victim than to confront a bully. This tendency might be explained by the lower risk to the bystander associated with reaching out to a victim or a preference for emotion-focused [70, 71] efforts to help a victim cope with cyberbullying. This would point to the relevance of the bystander intervention model [54] in the determination of which cyberbystander behaviors witnesses to cyberbullying may choose to enact and highlight a potentially meaningful distinction between victim-focused and bully-focused approaches to intervention [23, 69].

Given the robust likelihood of bystander inaction in our study, we found it interesting that the most indirect type of cyberbystander action—flagging or reporting a cyberbullying post—did not consistently emerge as the second most-likely response. In general, the likelihood of flagging a post did not exceed or was very close to other positive bystander behaviors, such as supporting the victim or confronting the bully. Future research to better understand this somewhat counterintuitive finding is warranted, given previous research indicating a greater likelihood of indirect compared to direct forms of cyberbystander behavior [25, 26]. It is possible that the average social media user may not be aware of how to flag or report harmful content on a given platform or may feel as though doing so will not result in any meaningful repercussions for the cyberbully. Whereas the use of at least one major social media platform was a criterion for inclusion in the present study, the sample—and social media users, broadly—may be less familiar with platform moderation policies and the concrete steps needed to engage with various flagging or reporting features. The limited use of flagging/reporting options could thus be an indicator that platform-based solutions alone may not be sufficient without proper training for the users. Alternatively, cyberbystanders may be especially skeptical about the effectiveness of flagging or reporting features for the provision of timely or meaningful support to a victim.

The only significant three-way interaction between the Big Five traits, cyberbullying severity, and cyberbystander behavior was for agreeableness. In response to high severity cyberbullying, participants high in agreeableness were more likely to confront the bully than to flag the post or reinforce the bully, whereas those lower in agreeableness did not display this tendency. In contrast, in response to low severity or non-cyberbullying instances, such differences were not observed. This pattern distinguishes agreeableness from conscientiousness and extroversion, suggesting that high agreeableness may be more dependent on situational factors and more directly associated with prosocial bystander behavior. Extroversion and conscientiousness, in contrast, were associated with variations in cyberbystander behavior but did not interact with severity level, suggesting that these traits predict relatively stable behavioral preferences that are less influenced by contextual factors such as cyberbullying severity.

The findings for agreeableness might also be interpreted through the lens of moral outrage and a heightened sensitivity to perceived injustice. In other words, because highly agreeable individuals are generally more concerned about others’ welfare and social harmony [72, 73], severe cyberbullying may be perceived as a violation of moral and social norms rather than a simple interpersonal conflict. In such cases, confrontation may function as a principled attempt to restore broader social harmony rather than as an aggressive response. This finding is also consistent with research suggesting that empathic anger or moral outrage can motivate bystanders to defend victims and confront perpetrators in cyberbullying contexts [74, 75].

We believe that the findings of this study have meaningful implications for the design of anti-cyberbullying interventions. Given that passive observation was consistently the most common response, educational programs should prioritize strategies to promote prosocial engagement from inactive bystanders. A complementary approach may be for platforms to incentivize active (prosocial) bystander intervention by seeking to shift the norms of the passive majority. Moreover, our results highlight that personality traits likely shape how individuals respond to cyberbullying under different conditions, suggesting that interventions may benefit from tailoring approaches to different personality profiles. For example, prior research has investigated the utility of “empathy nudges”—e.g., automated messages generated within a platform that prompt individuals to think about how other users may be feeling—for eliciting prosocial bystander behavior [26]. The effectiveness of these nudges, however, was limited. One intriguing possibility is that the effectiveness of subtle platform-generated prompts, like empathy nudges, might be further moderated by Big Five traits. Individual differences in traits could be extracted based on aspects of users’ social media activity, social network size, or actual content they have generated [71], which, in turn, could inform the activation of user-specific features. Overall, these insights emphasize the importance of more tailored and specific strategies in digital citizenship education to strengthen bystander intervention and reduce the harmful impact of cyberbullying.

The current findings also shed light on how technological affordances may relate to different types of cyberbystander behavior. To illustrate, Brody and Vangelisti [76] and DiFranzo et al. [26] have discussed the technical affordance factors of visual anonymity, visible co-presence, and audience scale as potential predictors of the likelihood that bystanders will intervene to support cyberbullying victims. You and Lee [77] proposed a nonlinear relationship between audience scale and probability of prosocial cyberbystander intervention, with an initially increasing and then decreasing likelihood of intervention as audience scale increases. Other types of affordances, such as privacy, visibility, editability, association, and searchability, have also been investigated as predictors of bystander intervention [78]. In the context of the present findings, it is possible that technological affordances may not only shape the likelihood of different cyberbystander behaviors but also interact with users’ internal traits in distinct ways.

### Limitations

This study had several methodological limitations that warrant discussion and elucidate important directions for future research. For instance, the present study relied exclusively on participants’ self-reported likelihood of performing different cyberbystander behaviors in response to ostensible instances of cyberbullying among anonymized strangers. A lack of self-awareness and social desirability concerns may, however, have led participants to over- or underreport their actual likelihood of performing certain cyberbystander behaviors. Moreover, the inclusion of additional decision-process indicators, such as response latencies or participants’ confidence in their responses, could have yielded valuable insights. To illustrate, future research could incorporate process-level indicators and apply computational approaches, such as Bayesian confidence models, to examine how cyberbullying severity shapes the latent decision-making processes underlying cyberbystander responses. Such models could help reveal whether more severe cyberbullying incidents increase evidence accumulation, strengthen decision confidence, or lower the threshold for active intervention [79].

Another set of limitations stems from the screenshots used as experimental stimuli in the present study. First, our key experimental stimuli were based exclusively on Instagram scenarios. Thus, participants’ degree of familiarity with the platform may have influenced how they interpreted and responded to the content. We did not, however, specifically assess participants’ Instagram use or platform familiarity, limiting our ability to account for this potential source of variability. Second, idiosyncratic effects arising from specific screenshots could have influenced the results. Whereas the use of profanity or content related to appearance-based humiliation within the screenshots could impact bystanders’ reactions, the potential for these effects was not explored in our analyses. Future research could investigate these effects by incorporating more cyberbullying interaction screenshots and performing analyses at the level of individual screenshots to better isolate the impact of specific contextual features. Third, screenshot order was randomized to prevent potential order effects, such as fatigue or sensitization, across conditions. Unfortunately, the exact order in which the screenshots were presented to each participant was not recorded by our survey software (Qualtrics). The inclusion of trial-level order data in future research will allow for the modeling of potential order effects.

Additionally, the screenshots used in this study only depicted dyadic interactions that may not have fully captured the more complex social dynamics of real-world social media environments. In actual online settings, cyberbullying often occurs within broader comment threads involving multiple commenters, passive audiences, likes, replies, and other visible cues of group behavior. These contextual features may shape perceived social norms, normative pressure, and bystanders’ willingness to intervene. Future research should therefore use stimuli that better simulate multi-user interactions and incorporate visible digital indicators of group responses, such as the number of likes or supportive or aggressive replies from users outside of the victim-bully dyad, to enhance ecological validity. A final limitation of the screenshots pertains to the conceptual ambiguity of “confronting User B” and “reinforcing User B” for the scenarios in which no cyberbullying was depicted. As a result, responses in the control condition may not be as directly comparable to those in the low severity and high severity cyberbullying conditions.

Lastly, we note limitations tied to the use of the Prolific.com survey platform. Although Prolific.com has been found to yield relatively high-quality data compared to other online survey platforms [80], prior research has noted potential threats to data quality in online samples, including bots and duplicate participants [81, 82]. Other limitations stem from the demographic composition of our Prolific.com sample. For example, our sample predominantly comprised White and heterosexual participants, indicating an underrepresentation of racial and sexual minority groups. Given that minority groups are often more susceptible to cyberbullying [2, 83, 84], their experiences merit particular attention in subsequent research. The present study also focused exclusively on adults, leaving the behavior of adolescent bystanders unexplored, even though cyberbullying may be more common and especially harmful among adolescents [2]. The older participants in our sample, in particular, may have been more inclined to remain a passive observer given difficulty relating to the (younger) developmental life stage of the users depicted in the screenshots. To help control for this, we included age as a covariate in all linear mixed effects models and, notably, age was not a significant correlate of any of the cyberbystander behaviors we assessed. Yet, this remains an interesting avenue to explore in future studies.

## Conclusion

This research provides initial experimental evidence of the potential importance of investigating personality traits and their interaction with cyberbullying severity within the context of cyberbystander behavior. Although it demonstrates a robust tendency toward passive observation on the whole, it also points to the predictive value of Big Five personality traits and understanding contextual factors in cyberbullying instances, including severity, in cyberbystander research. We hope that the findings can help advance the emerging literature on cyberbystander behavior and contribute to building more effective anti-cyberbullying features on social media platforms.

## Supplementary Information

Supplementary Material 1

## Data Availability

The datasets used and/or analysed during the current study are available from the corresponding author on reasonable request.
