# Supplementary material for: Big Five traits and cyberbullying severity as predictors of cyberbystander behavior among adults in the U.S
Source: BMC Psychol. 2026 Jul 17;14:1297. doi: 10.1186/s40359-026-05073-5 (PMC13551842; doi:10.1186/s40359-026-05073-5)
Supplement: Supplementary file 1 [file 40359_2026_5073_MOESM1_ESM.docx]

User A Follow

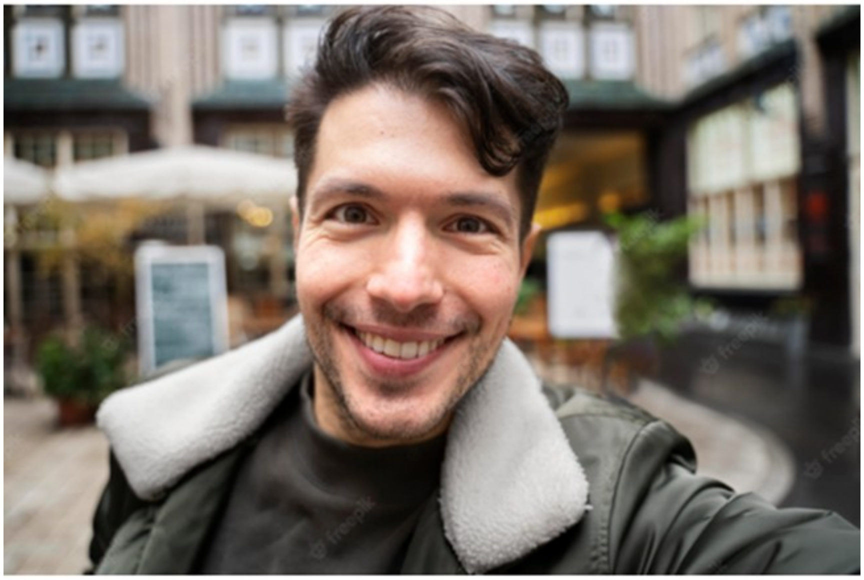

[illegible] likes

User A Hanging out!

User B Those huge ugly buck teeth are not doing you any favors buddy

User A @K[illegible]s Seriously? u know if u have nothing nice to say...

User B @E[illegible]n not to you f*** ugly loser

Add a comment...

50 days ago

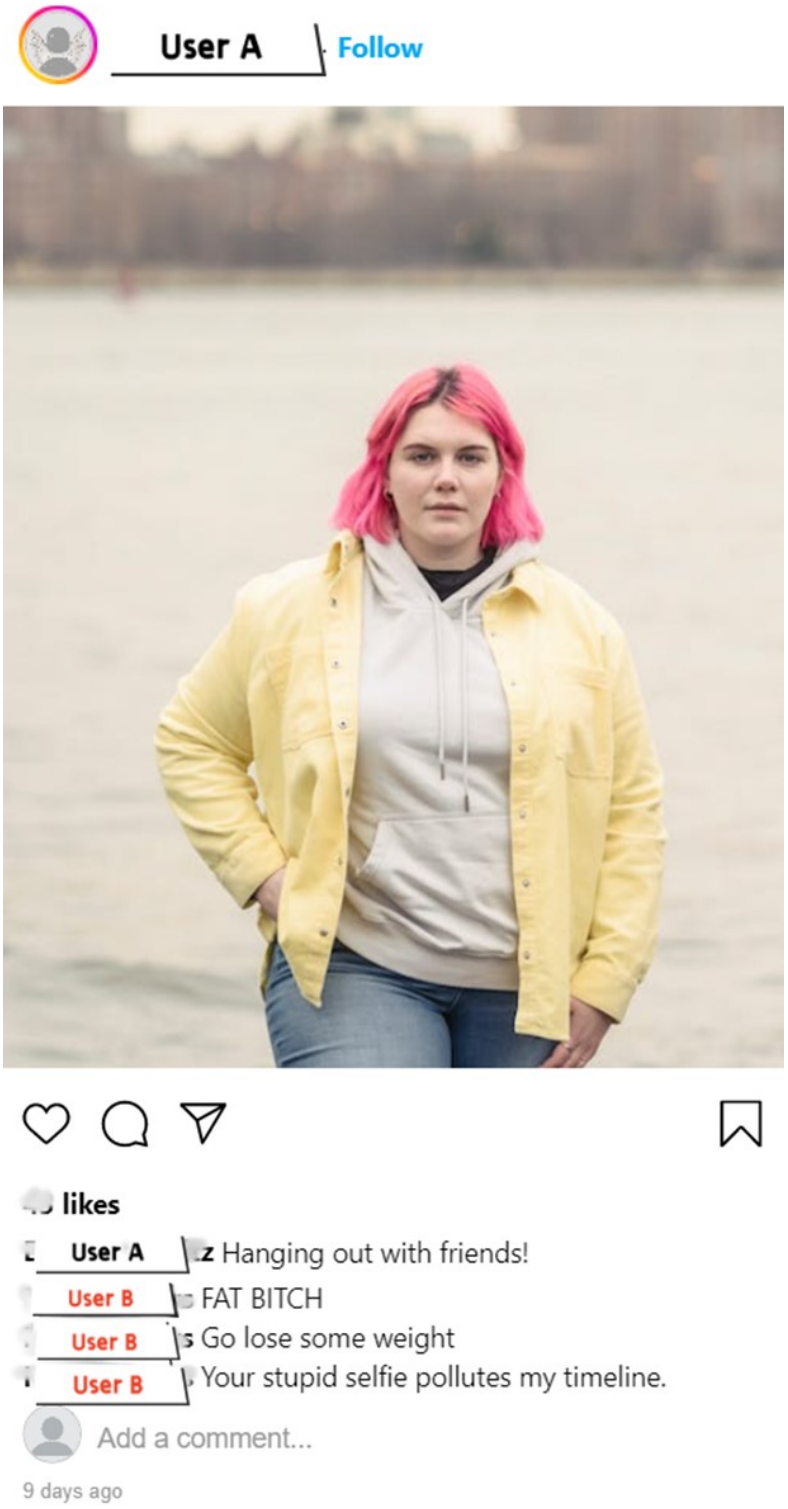
User A
Follow
likes
User A Hanging out with friends!
User B FAT BITCH
User B Go lose some weight
User B Your stupid selfie pollutes my timeline.
Add a comment...
9 days ago

User A · Follow

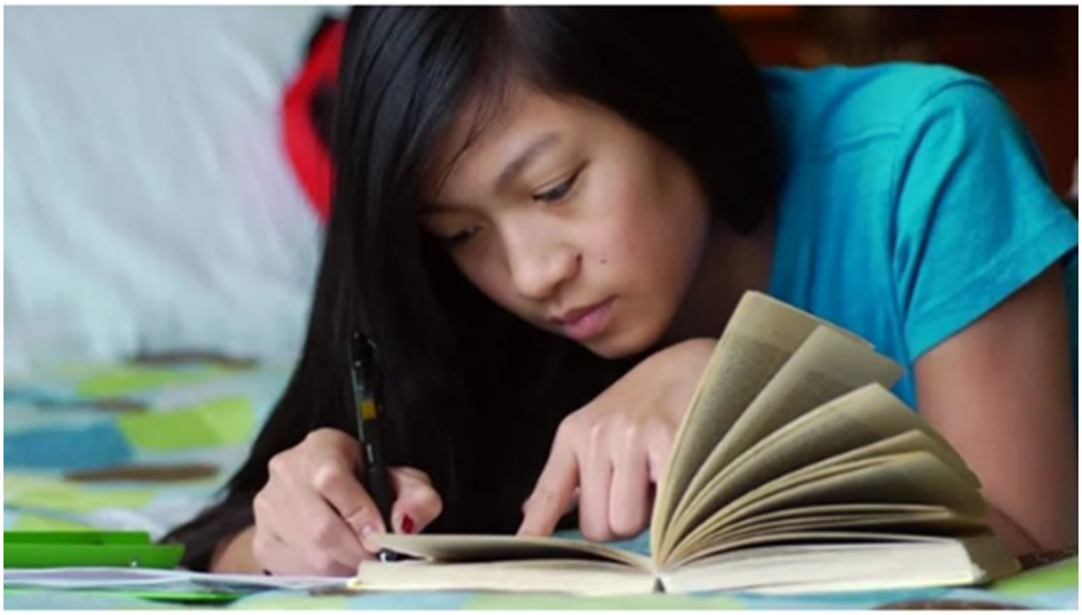

[illegible] likes

User A Catching up on some studying!

User B You practically failed Mr. Wrigley's class.

User B LOSER

User B Your an idiot dont pretend

Add a comment...

14 days ago

UserA · Follow

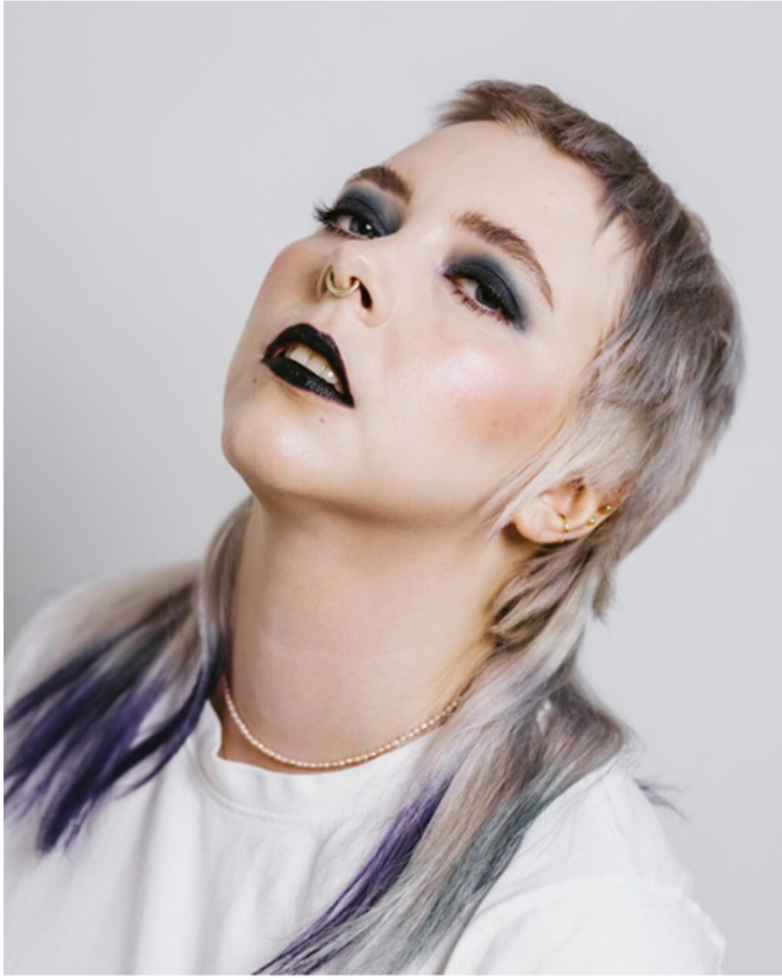

[illegible] likes

UserA s new hair don't care:)

UserB a mullet really? is it the 80's?

UserA s @[illegible]t cool opinion keep it to yourself

UserB @[illegible] whatever just saying.

Add a comment...

36 days ago

User A Follow

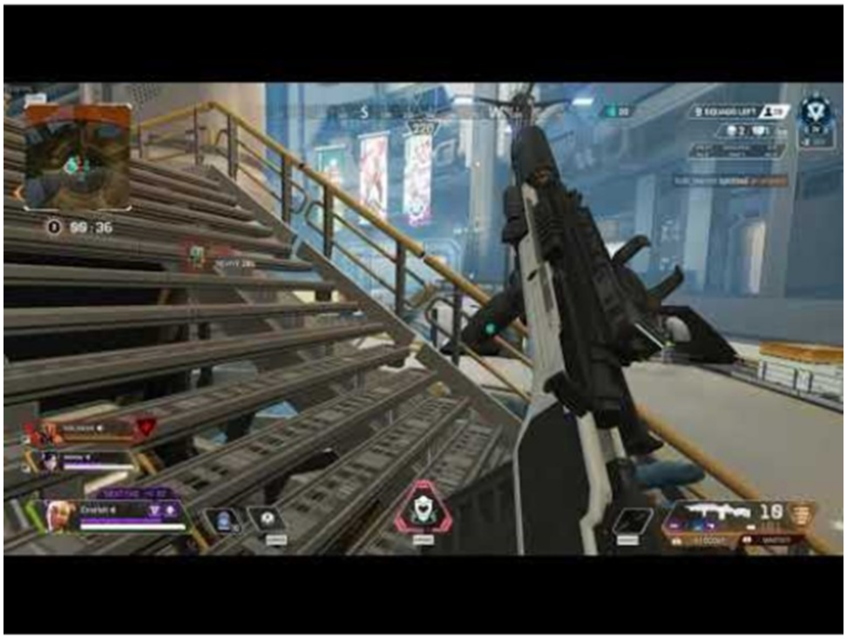

[illegible] **likes**

User A The shooting feel of APEX is relatively poor. Game designer should optimize that.

User B It's because of you. Apologize to the game!

User A @[illegible] What are you saying?

User B @[illegible] This game is only for smart people. You are not one of us.

Add a comment...

5 days ago

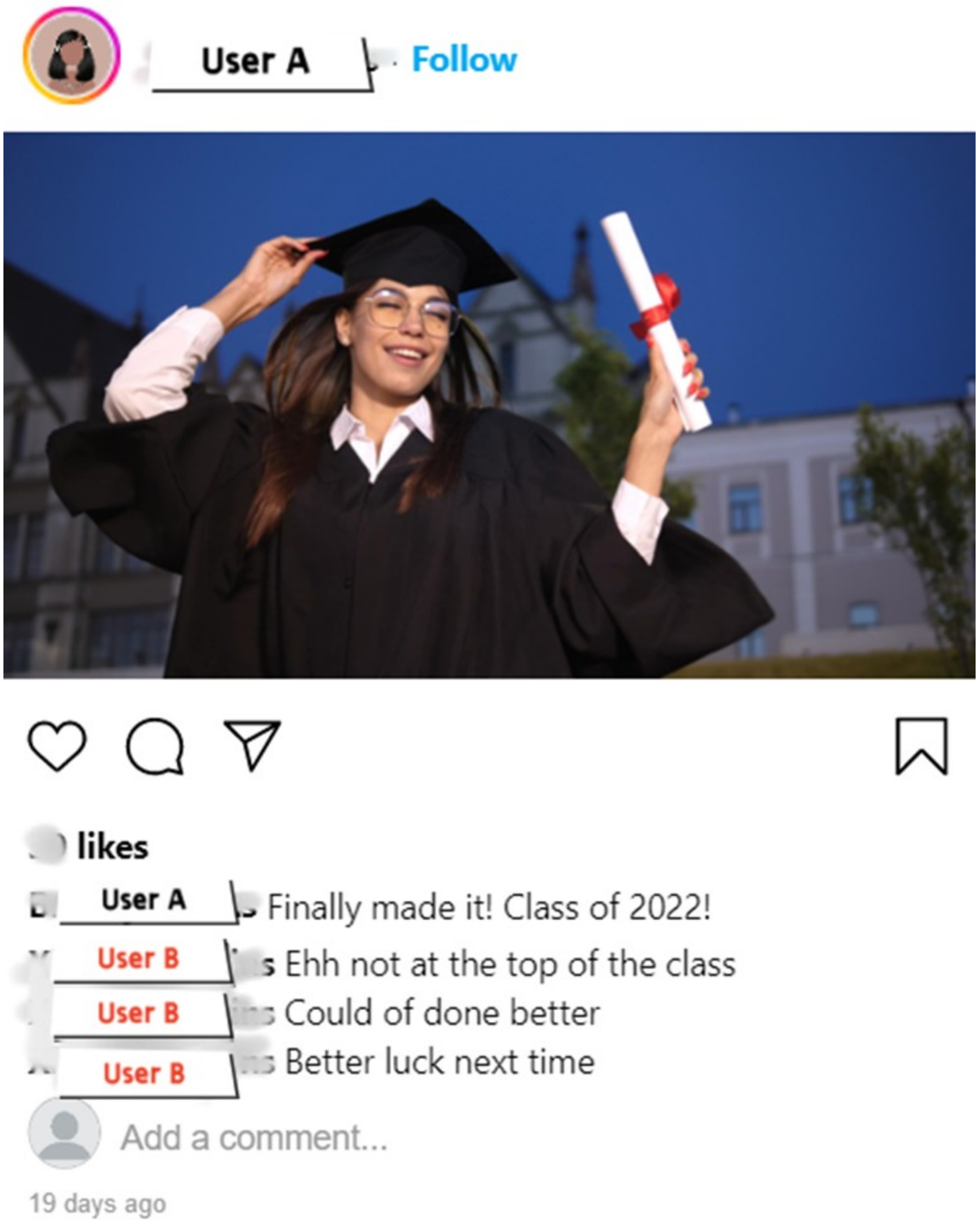
User A
Follow
likes
User A Finally made it! Class of 2022!
User B Ehh not at the top of the class
User B Could of done better
User B Better luck next time
Add a comment...
19 days ago

User A · Follow

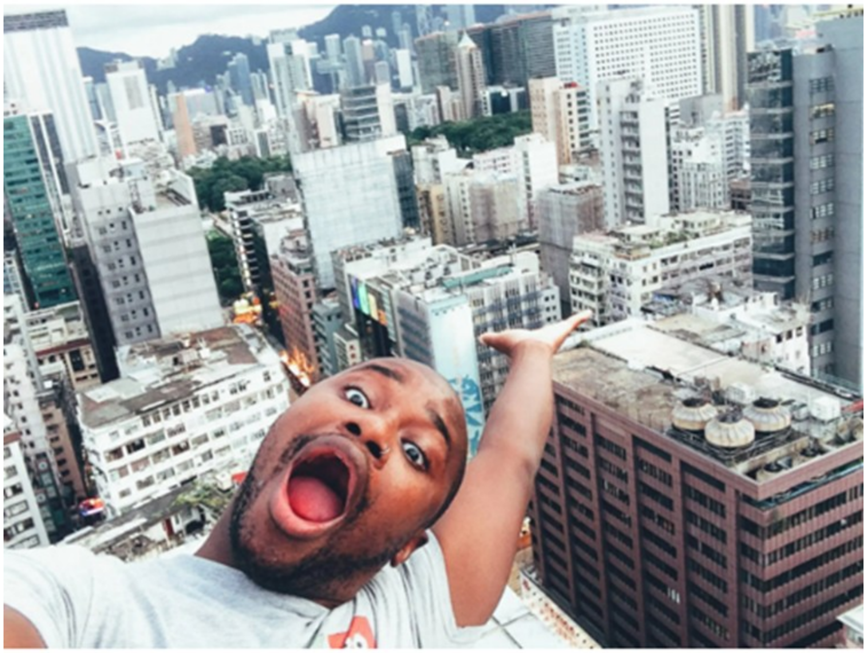

[illegible] likes

User A [illegible] new york, new york baby!!

User B [illegible] nice view bro!

User A [illegible] @J[illegible]n thanks man it's awesome here

User B [illegible] @H[illegible]n looks it!

Add a comment...

22 days ago

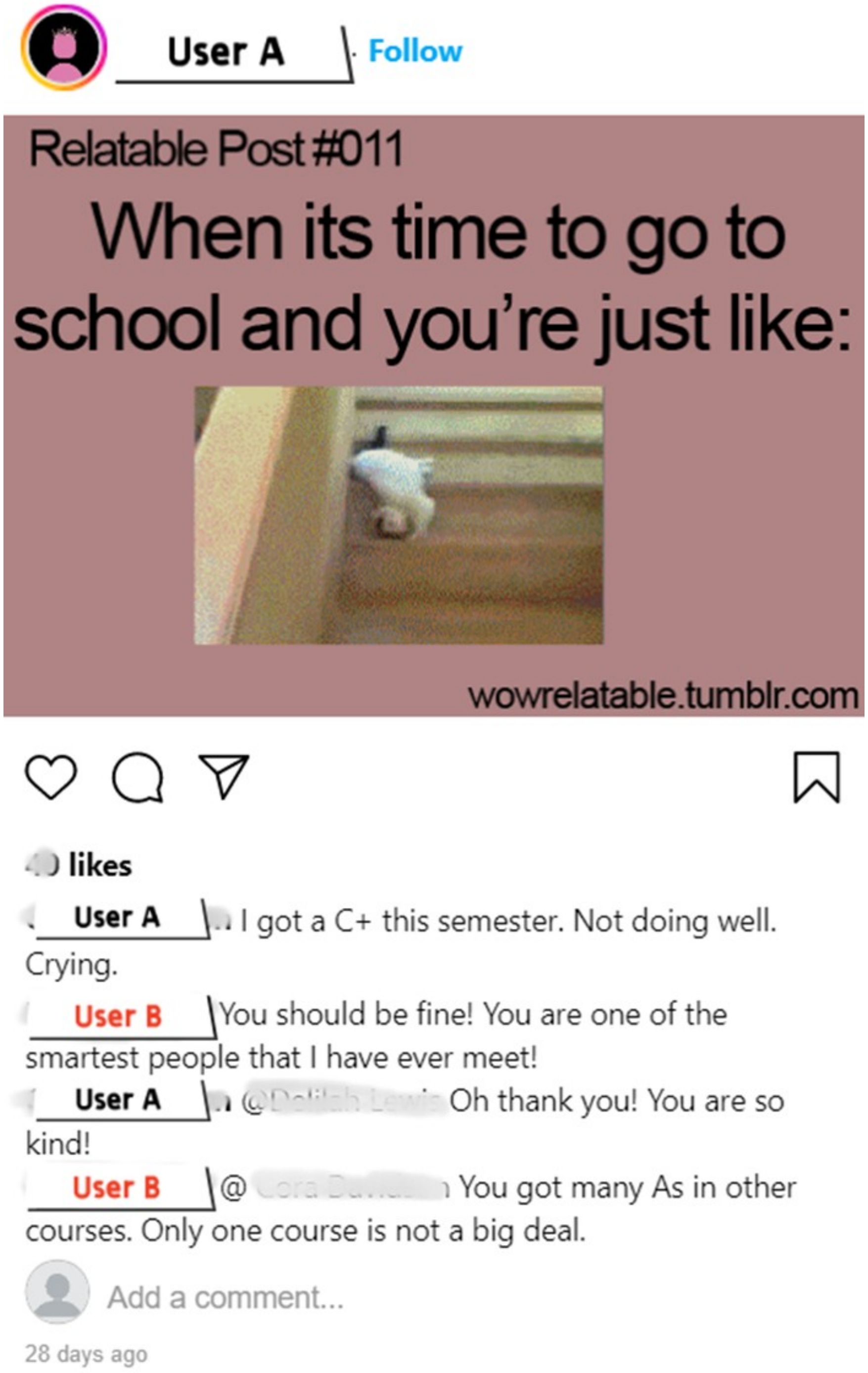
User A
Follow
Relatable Post #011
When its time to go to school and you’re just like:
wowrelatable.tumblr.com
likes
User A I got a C+ this semester. Not doing well. Crying.
User B You should be fine! You are one of the smartest people that I have ever meet!
User A Oh thank you! You are so kind!
User B @ You got many As in other courses. Only one course is not a big deal.
Add a comment...
28 days ago

User A Follow

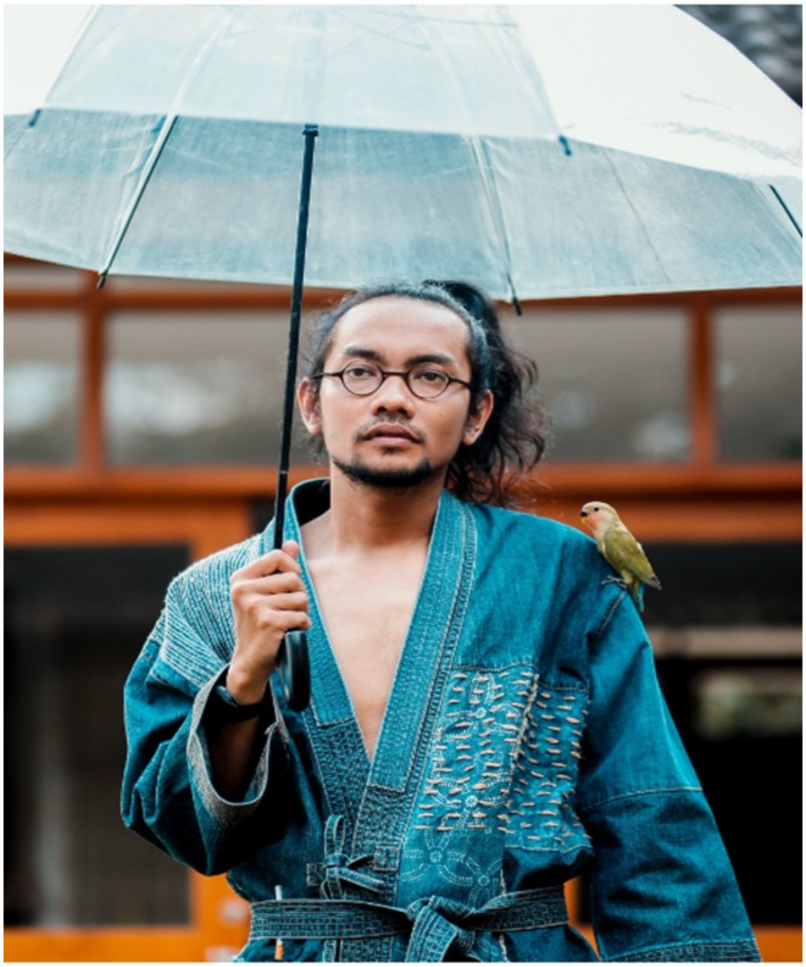

[illegible] **likes**

User A Traveling in Japan.

User B I like your haircut bro

User B You look smart in this kimono!

User B Oh there's a parrot on your shoulder!

Add a comment...

7 days ago
